# Integrated omics approaches provide strategies for rapid erythromycin yield increase in *Saccharopolyspora erythraea*

**DOI:** 10.1186/s12934-016-0496-5

**Published:** 2016-06-03

**Authors:** Katarina Karničar, Igor Drobnak, Marko Petek, Vasilka Magdevska, Jaka Horvat, Robert Vidmar, Špela Baebler, Ana Rotter, Polona Jamnik, Štefan Fujs, Boris Turk, Marko Fonovič, Kristina Gruden, Gregor Kosec, Hrvoje Petković

**Affiliations:** Acies Bio, d.o.o., Tehnološki park 21, SI-1000, Ljubljana, Slovenia; Department of Biotechnology and Systems Biology, National Institute of Biology, Večna pot 111, SI-1000, Ljubljana, Slovenia; Department of Biochemistry, Molecular and Structural Biology, Jožef Stefan Institute, Jamova cesta 39, SI-1000, Ljubljana, Slovenia; Department of Food Science and Technology, Biotechnical Faculty, University of Ljubljana, Jamnikarjeva 101, SI-1000, Ljubljana, Slovenia; International Postgraduate School Jožef Stefan, Jamova cesta 39, SI-1000, Ljubljana, Slovenia; Faculty of Chemistry and Chemical Technology, University of Ljubljana, Aškerčeva cesta 5, SI-1000, Ljubljana, Slovenia; Centre of Excellence for Integrated Approaches in Chemistry and Biology of Proteins, Jamova cesta 39, SI-1000, Ljubljana, Slovenia; Department of Synthetic Biology and Immunology, National Institute of Chemistry, Hajdrihova 19, SI-1000, Ljubljana, Slovenia

**Keywords:** *Saccharopolyspora erythraea*, Erythromycin, Systems biology, Metabolic engineering, Transcriptomics, Proteomics, Polyketide, Substrate supply

## Abstract

**Background:**

Omics approaches have significantly increased our understanding of biological systems. However, they have had limited success in explaining the dramatically increased productivity of commercially important natural products by industrial high-producing strains, such as the erythromycin-producing actinomycete *Saccharopolyspora erythraea*. Further yield increase is of great importance but requires a better understanding of the underlying physiological processes.

**Results:**

To reveal the mechanisms related to erythromycin yield increase, we have undertaken an integrated study of the genomic, transcriptomic, and proteomic differences between the wild type strain NRRL2338 (WT) and the industrial high-producing strain ABE1441 (HP) of *S. erythraea* at multiple time points of a simulated industrial bioprocess. 165 observed mutations lead to differences in gene expression profiles and protein abundance between the two strains, which were most prominent in the initial stages of erythromycin production. Enzymes involved in erythromycin biosynthesis, metabolism of branched chain amino acids and proteolysis were most strongly upregulated in the HP strain. Interestingly, genes related to TCA cycle and DNA-repair were downregulated. Additionally, comprehensive data analysis uncovered significant correlations in expression profiles of the erythromycin-biosynthetic genes, other biosynthetic gene clusters and previously unidentified putative regulatory genes. Based on this information, we demonstrated that overexpression of several genes involved in amino acid metabolism can contribute to increased yield of erythromycin, confirming the validity of our systems biology approach.

**Conclusions:**

Our comprehensive omics approach, carried out in industrially relevant conditions, enabled the identification of key pathways affecting erythromycin yield and suggests strategies for rapid increase in the production of secondary metabolites in industrial environment.

**Electronic supplementary material:**

The online version of this article (doi:10.1186/s12934-016-0496-5) contains supplementary material, which is available to authorized users.

## Background

Metabolic engineering of microorganisms for industrial production of valuable compounds has been influenced strongly in recent years by the growing availability of “omics” techniques. Genomics and transcriptomics, but also proteomics and metabolomics have importantly increased our systems-wide understanding of cell physiology, from transcriptional and translational regulation to morphogenesis, stress response and many other processes in the cell [1]. In many cases, particularly for production of metabolites with well-characterized biosynthetic pathways in well-known microbes, results of omics analyses have inspired novel metabolic engineering strategies which led to significant improvements of titres of industrially important metabolites such as l-lysine, l-threonine or xylitol [2–5]. In addition, integration of different types of omics data through systems biology approaches is a very promising tool in process development which will further accelerate the development of efficient bioprocesses for production of natural products as well as non-natural chemicals [6].

Soil-dwelling actinomycete bacteria are particularly prolific producers of diverse biologically active secondary metabolites, which have found widespread use in human and veterinary medicine, as well as in agriculture [7]. These substances include antibiotics, antifungal, anti-cancer, immunosuppressive, insecticide, and other classes of bioactive substances of immense value for human health and the global economy. In natural environments these compounds are produced by bacterial cells in very small amounts as non-essential secondary metabolites. Decades of research have been devoted into improvement of diverse strains and fermentation bioprocesses used to produce these metabolites at industrial scale. Predominantly, strains were subjected to random mutagenesis treatments followed by intensive strain selection aiming at improved yields of target products [8, 9]. As a result, many industrially used strains now have drastically, up to 1000 fold, improved secondary metabolite yields. However, they are also full of uncharacterized mutations that disrupt their normal developmental cycle, make them more sensitive to environmental conditions, and hinder further yield optimization.

Omics methods are clearly opening new possibilities to, more rapidly than before, turn actinomycetes into sophisticated and efficient cell factories. Better understanding of their physiology will enable researchers to selectively remove the metabolic bottlenecks of secondary metabolite biosynthesis without introducing unnecessary or deleterious changes. A “reverse engineering” approach has been proposed, whereby factors important for production could be identified by examining the differences between natural strains and industrial high-producing strains obtained by random mutagenesis and selection. Impressive insight into actinomycete biology has already been achieved by omics techniques [10]. For example, genome sequencing revealed an unimagined potential of actinomycete strains to synthesize a large number of different bioactive compounds [10]. However, mechanisms responsible for increasing the yields of valuable bioactive compounds are still poorly understood and omics approaches have shown relatively limited success in improving production of important natural products at industrial scale. This is likely due to several reasons: (1) actinomycetes have large genomes, up to 12 Mbp, which is reflected in complex networks of global and pathway-specific regulatory genes and morphological differentiation [1, 11]; (2) the interrelations between primary and secondary metabolic pathways during the production stage of the bioprocess are complex and poorly understood [12]; (3) laboratory media, in which transcriptomic and proteomic analyses are usually carried out, poorly reflect industrially relevant bioprocess conditions, whereas omics analyses in rich industrial media are hampered by difficulties in isolating high quality RNA and/or proteins; (4) most industrially used actinomycete strains have likely been “over-mutagenized” during the strain improvement process, resulting in a large number of observed genomic variants (SNPs), many of which represent neutral or even negative mutations, often resulting in morphologically and physiological unstable strains, which are difficult to manage reproducibly in industrial environment [13]. All these factors make application of omics analyses and their interpretation even more complex for industrial actinomycetes than for most other microbes.

*Saccharopolyspora erythraea* is a particularly interesting organism to study, as the producer of erythromycin, an industrially and clinically extremely valuable antibiotic, but also as a model representative of actinomycetes [14]. Since publication of the *S. erythraea* genome [15], several studies have investigated the changes in gene expression throughout the erythromycin production bioprocess, in both wild type and industrial strains [16–21]. Despite the progress made in these studies, much work remains to be done in assigning functional roles to mutations found in industrial *S. erythraea* strains, identifying key mechanisms influencing erythromycin yield and clarifying the connections between erythromycin biosynthesis and the rest of cellular metabolism. In order to enable rapid increase of erythromycin yield by metabolic engineering/synthetic biology approaches, much deeper understanding of these aspects is of great importance.

In an effort to address some of these questions, we have undertaken a comprehensive comparative study of the genomic, transcriptomic, and proteomic differences between the wild type *S. erythraea* NRRL2338 (WT) and an industrial high-producing (HP) strain of *S. erythraea* ABE1441, which had been subjected to mutagenesis and selection for many decades. Importantly, cultivation of both strains was carried out at bioreactor scale, using industrially relevant growth media and bioprocess parameters. Using various data analysis and integration approaches we identified several novel mechanisms that could contribute to higher erythromycin yield in the HP strain. We observed the overexpression of several genes related to branched-chain amino acid metabolism, potentially representing a novel methylmalonyl-CoA building block feeder pathway. Significant increase in final erythromycin yield was observed, when several of these genes were constitutively overexpressed in the WT strain. Our work shows that omics approaches can rapidly provide new strategies for the improvement of actinomycete based production strains, provided that the analyses are carried out with optimised methodology in industrially relevant conditions.

## Results

### Genome of the high-producing *S. erythraea* strain ABE1441

The order-of-magnitude increase in erythromycin yield, displayed in industrial cultivation conditions by the HP strain [22] compared to WT, as well as all differences in the metabolism between the two strains ultimately stem from genomic mutations. These mutations accumulated in the HP strain in numerous rounds of “classical strain” improvement by random mutagenesis and selection. Therefore, we initially sequenced the genome of the HP strain and compared it to the genome of the publicly available WT *S. erythraea* strain. Importantly, before comparison with the HP sequence, the originally deposited WT sequence [15] was screened for potential sequencing errors by comparing it with recently published RNA-seq data of the same NRRL 2338 strain [19]. Using this approach 40 putative sequencing errors were identified in the original WT genome of approx. 8.2 Mbp (Additional file 1). Since these sequencing errors would falsely appear as “mutations” when comparing the HP strain to the published WT genome, they were excluded from our comparative genomic analysis. Next generation sequencing of the HP strain revealed 165 genuine mutations compared to WT, affecting 147 genes (i.e. present inside the ORFs or in putative promoter or terminator regions). Out of these, 139 were single nucleotide variations (SNVs), 23 multiple nucleotide variations (MNVs), two deletions (in terminator of SACE_5310 and in SACE_6447) and one insertion (in SACE_4589). Seven genes had two mutations while five and seven mutations were identified in two transposase genes SACE_3579 and SACE_4072, respectively. Mutations were found to be distributed evenly over the whole genome. Particularly noteworthy are mutations in three genes involved in the TCA cycle (SACE_1170, SACE_6584 and SACE_6636), three genes belonging to two-component regulatory systems (SACE_4067, SACE_6086, and SACE_6447) and seven genes involved in amino acid metabolism (SACE numbers 3033, 3126, 3584, 4093, 4116, 6565 and 7125). Locations and nature of all observed mutations are presented in Additional file 2, columns H-M.

### Adapted industrial-like bioprocess for erythromycin production

**Fig. 1:**
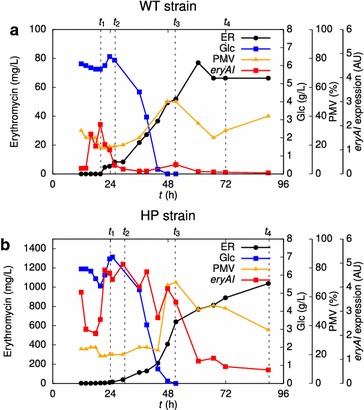
Key bioprocess parameters of cultivation of WT (**a**) and HP (**b**) strains in bioreactor scale. Erythromycin (*ER*) and glucose (*Glc*) concentrations, the packed mycelium volume (*PMV*), and relative expression of key erythromycin biosynthetic gene *eryAI* are plotted against total bioprocess time for a representative replicate of the bioprocess. The *dashed lines* denote the time points selected for transcriptomic and proteomic analysis. In the WT strain, erythromycin is produced primarily during the period of intense cell growth and rapid glucose consumption. In the HP strain, final erythromycin yield is much higher due to an increased rate of production during the rapid growth phase and a prolonged period of production in the late stages of the bioprocess. Expression profiles of *eryAI* and *eryK* erythromycin biosynthesis marker genes in both bioprocesses are shown in Additional file 3

In order to obtain industrially relevant transcriptomic and proteomic data, three independent bioprocesses with WT and HP strains were carried out in 5 L bioreactors and samples were taken at specific time points, characterized by rates of biomass growth, erythromycin titre, and expression of erythromycin biosynthetic genes (Fig. 1; Additional file 3). Briefly, the fermentation process consists of an initial phase without erythromycin production, lasting around 24 h (time point t1—initial increase in expression of the erythromycin biosynthetic genes *eryAI* and *eryK*; see Additional file 3). The initial phase is followed by a period of rapid biomass formation and rapid consumption of glucose, when erythromycin production is most pronounced (time point t2—start of rapid growth phase and detectable erythromycin production, transient decline of erythromycin biosynthetic gene expression). Once all the glucose in the medium is consumed and the cells enter the stationary growth phase (time point t3—glucose depleted, a second increase in expression of *eryA1* and *eryK* genes), there is an additional increase in erythromycin titre. However, the biosynthesis of erythromycin stops shortly afterwards in the WT strain (time point t4–stationary phase, end of erythromycin production in WT). In the HP strain, in contrast, erythromycin production is not only more intense during the rapid growth phase, but it also continues into the stationary phase, resulting in a more than ten-fold higher yield compared to the WT strain. Accordingly, prolonged expression of *eryAI* gene, encoding the erythromycin polyketide synthase, was also observed in the HP strain. Interestingly, the rate of glucose consumption is almost identical for both strains.

### Comparative gene expression analysis

**Fig. 2:**
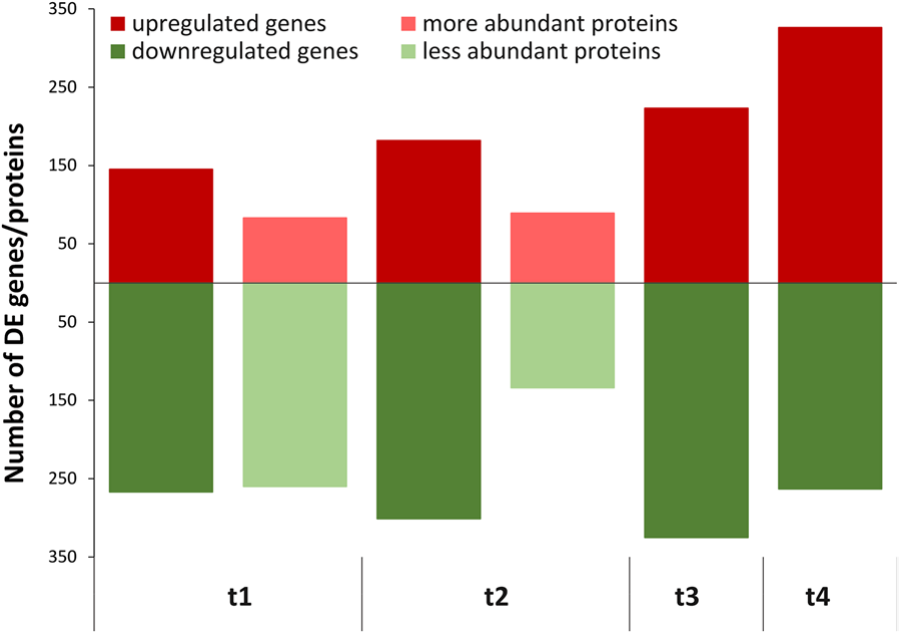
Numbers of differentially expressed genes and differentially abundant proteins at the four bioprocess time points. The numbers of up-regulated genes/proteins in HP compared to WT strain at the time points t1 to t4 of the fermentation process are shown in *red* and down-regulated in* green*. *Darker colour* is used for microarray analyses of gene expression and *lighter colour* for LC–MS/MS analyses of protein abundance. The cut-off for differential expression was |logFC| > 1 and p < 0.01 and for differential protein abundance was |logFC| > 1.5, respectively. Due to extensive proteolysis in the HP strain samples differential protein abundance was not calculated at t3 and t4

The samples obtained at defined time points of the adapted industrial bioprocess allowed us to conduct a thorough comparative analysis between WT and HP strains at the gene expression level. Firstly, we identified differentially expressed genes at individual time points. Out of 7217 genes featured on the microarray, 55 were excluded from subsequent data analysis due to low signal in most samples and 1198 genes were found to be significantly differentially expressed (DE) between HP and WT in at least one time point. The number of DE genes increased over time (Fig. 2). Microarray data was validated by performing qPCR analyses, based on seven selected genes related to erythromycin biosynthesis (Additional file 4). The high correlation between the results of the two methods (r = 0.84) confirms that the microarray gene expression data is reliable.

In the next step we performed a gene set enrichment analysis (GSEA) [23] to identify the functional gene groups and metabolic pathways (as defined by KEGG pathway or Gene Ontology) that were over- or under-expressed in the HP strain at selected time points during the fermentation (Table 1; Additional file 5). While some differences in gene expression between the strains can already be observed at t1, the switch to secondary metabolism at t2 is more pronounced in the HP strain, suggesting that in the HP secondary metabolism is turned on earlier than in the WT strain. This is seen as enrichment of upregulated genes in several secondary metabolic pathways and in pathways related to the metabolism of amino acids that can act as polyketide precursors, as well as by the enrichment of downregulated genes in primary metabolic pathways such as the TCA cycle and nucleotide and nitrogen metabolism. The HP strain then maintains the significantly higher expression of erythromycin biosynthetic genes as well as other polyketide-related pathways throughout the fermentation. Interestingly, the analysis shows that at t3, genes involved in DNA repair mechanisms during replication are expressed at lower level in HP compared to WT strain. Moreover, increased proteolysis, observed in proteomics, is also evident on the transcriptome level (Additional file 5). Overall, in comparison to WT, the HP strain shows increased expression of genes involved in secondary metabolism and decreased expression of several key primary metabolic pathways since the start of the exponential growth phase (t2). This becomes even more pronounced once the glucose in the medium is fully consumed (t3) (Fig. 1).

**Table 1:** Transcriptional differences in HP compared to the WT strain at the level of biological processes

Gene sets				HP vs WT			
Ontology	Name	Code	Size	t1	t2	t3	t4
KEGG Pathway	Translation		145	+	+		
	Ribosome	sen03010	64	+	+		
	Metabolism of terpenoids and polyketides		128		+	+	+
	Biosynthesis of ansamycins	sen01051	13		+	+	+
	Biosynthesis of 12-, 14- and 16-membered macrolides	sen00522	8		+	+	+
	Type I polyketide structures	sen01052	9		+	+	+
	Nitrogen metabolism	sen00910	56		−		
	C5-branched dibasic acid metabolism	sen00660	10		+		
	Valine, leucine and isoleucine biosynthesis	sen00290	23		+		
	Tyrosine metabolism	sen00350	38	−			
	Tryptophan metabolism	sen00380	39			+	+
	Glycolysis/gluconeogenesis	sen00010	74		−		
	Citrate cycle (TCA cycle)	sen00020	48		−		
	Pyruvate metabolism	sen00620	73		−		
	Metabolism of cofactors and vitamins		192		+		
	Lipid metabolism		169	−			
	Fatty acid biosynthesis	sen00061	29	−			
	Xenobiotics biodegradation and metabolism		175	−			
	Styrene degradation	sen00643	13			+	
	Atrazine degradation	sen01052	9	−	−		
	Purine metabolism	sen00230	88		−	−	
	Nucleotide metabolism		118		−	−	−
	Oxidative phosphorylation	sen00190	49	+			
	Inositol phosphate metabolism	sen00562	20			+	
	Membrane transport		138	−			
	ABC transporters	sen02010	117	−			
GO biological process	DNA biosynthetic process	GO:0071897	18		−	−	
	DNA replication	GO:0006260	29			−	−
	DNA integration	GO:0015074	48				−
	DNA recombination	GO:0006310	44	+			−
	DNA repair	GO:0006281	45			−	
	Transposition, DNA-mediated	GO:0006313	62		−	−	−
	DNA-templated transcription, initiation	GO:0006352	36	−	−	−	
	Negative regulation of transcription, DNA-templated	GO:0045892	19		−	−	
	Translation	GO:0006412	68	+	+		+
	Methylation	GO:0032259	126			+	
	Dephosphorylation	GO:0016311	26			+	
	Biosynthetic process	GO:0009058	59			+	+
	Fatty acid biosynthetic process	GO:0006633	23	−			
	Antibiotic biosynthetic process	GO:0017000	16		+	+	+
	Proteolysis	GO:0006508	152		+	+	+
	Amino acid transmembrane transport	GO:0003333	33	−			
	Carbohydrate transport	GO:0008643	25				−

For each ontology used, KEGG pathways or GO Biological process, gene set names, codes and sizes (total numbers of *S. erythraea* genes associated with the gene set) are shown. Significant enrichment (p < 0.01 for KEGG and p < 0.05 for GO) of the individual gene sets in up- or down-regulated genes at individual time points (t1–t4) is represented by ‘+’ or ‘−’, respectively. Pathways with missing codes are the top KEGG ontology terms

Complementary to analyses described above where only differences in individual time points were assessed, time-course profile analysis was performed that identified 6 clusters of genes with differential time-course profiles between the two strains (Additional file 6). In two clusters the gene expression was higher and in 4 lower in WT compared to HP throughout the fermentation process.

### Co-expression analysis

**Fig. 3:**
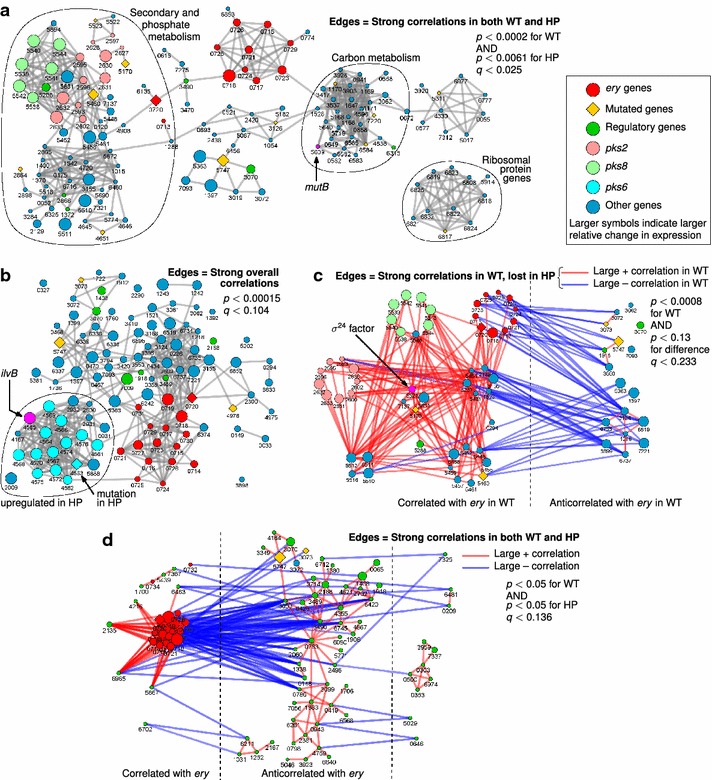
Graph representations of networks of genes with correlated expression profiles.* Nodes* represent genes, identified by their SACE numbers, and *edges* are drawn between genes whose expression levels show a strong (positive or negative) correlation in different contexts. **a** Edges are drawn between genes whose expression levels show a strong correlation in the WT strain and maintain the same correlation in the HP strain. Only genes closely correlated to at least one mutated gene (p < 0.00017 in WT) are shown. The *ery* cluster is connected to both the primary carbon metabolism (including *mutB*/SACE_5639, *highlighted in magenta*) and secondary metabolic genes. A separate cluster of ribosomal proteins was also identified using this approach, but it is not correlated to *ery* genes. **b** Edges are drawn between genes whose expression is strongly correlated in the full dataset, across both strains. Only genes with a strong correlation to the *ery* cluster (top 6 % of all genes) and a large change in expression between WT and HP strains (top 3.7 %) are shown. Genes of the *pks6* cluster (SACE_4561-76) form a strongly interconnected group that is overexpressed in HP and includes a mutated gene (SACE_4563) and *ilvB* (SACE_4565, *highlighted in magenta*). **c** Edges are drawn between genes that are strongly correlated in the WT strain but whose correlation is greatly perturbed in the HP strain. Only genes with a high WT correlation to at least one mutated gene (p < 0.00016) and with a large change in expression between HP and WT (top 3.7 %) are shown. The putative sigma24 factor (SACE_5521, *highlighted in magenta*) loses many positive correlations to *pks2* and *pks8* genes. **d** Correlations between the *ery* cluster and regulatory genes. Edges are drawn between genes that maintain strong correlations in both strains. Genes consistently correlated with *ery* represent potential activators and those anticorrelated represent potential repressors

In the next step we also investigated the correlations between gene expression levels [24, 25]. For each gene in the genome we calculated Pearson correlation coefficients between the expression levels of that gene and all other genes across all experimental samples. The same analysis was also performed on subsets of data limited to the WT strain and the HP strain, respectively. Our analysis revealed that the *ery* cluster is highly correlated to a wide range of genes encoding diverse functions (Fig. 3). At least two distinct groups of genes are consistently correlated with the expression of the *ery* cluster in both WT and HP strains while also having a strong connection to at least one mutated gene (Fig. 3a). The first group contains a number of genes of the TCA cycle and fatty acid metabolism, as well as *mutB* (SACE_5639, a member of the *mcm* operon involved in converting between succinyl- and methylmalonyl-CoA). These genes are expected to be involved in the supply of precursors for erythromycin biosynthesis from the core carbon metabolism. The second group is mostly composed of genes related to the phosphate metabolism or to the biosynthesis of secondary metabolites. The latter include a pair of apparent polyketide synthase (PKS) clusters, *pks2* (SACE_2593-635) and *pks8* (SACE_5532-45). Interestingly, even though their expression is much lower in HP than in WT, these PKS genes maintain a positive correlation with the *ery* cluster across the time points of WT as well as HP fermentation. This suggests they may be subject to dual regulation where one system strives to coordinate their expression to that of *ery*, while a separate mechanism strongly downregulates their expression in HP. Our analysis also identified other highly internally connected groups of genes corresponding to ribosomal proteins and several transposons (Fig. 3a), confirming that the expression correlation approach can successfully identify co-regulated genes.

We also examined genes that are globally regulated (across both strains and all time points) in concert with *ery* and, simultaneously, are dramatically over-or under-expressed in the HP strain (Fig. 3b). This includes a tightly interconnected cluster of genes (SACE_4561-76) whose expression does not correlate very strongly with the *ery* cluster in WT, but closely mirrors *ery* overexpression in the HP strain. These genes were also identified in the same cluster as *ery* genes in the regression-based time-course profile analysis (Additional file 6). They are annotated as encoding an “alternative” polyketide synthase system, *pks6*, along with an acetolactate synthase (*ilvB1*, SACE_4565) and a valine-pyruvate transaminase (*avtA*, SACE_4564) that presumably ensure a steady supply of precursor metabolites. Importantly, one of the *pks6* genes (SACE_4563, unknown function) is mutated in the HP strain, which may block the actual synthesis of the corresponding polyketide product.

Finally, we focused on genes that have both their expression levels as well as their coordination/correlations with other genes strongly perturbed in the HP strain (Fig. 3c). This group includes several genes of the PKS clusters *pks2* and *pks8*, as well as a putative sigma24 factor (SACE_5521) with strong correlations to both of these PKS clusters in the WT strain. All three are strongly downregulated in the HP strain. In addition, the most prominent regulatory proteins that correlate strongly with the *ery* cluster in either or both strains are examined further in Fig. 3d and Additional file 7. Several regulators are co-expressed with *ery* and others show the opposite expression pattern (negative correlation). Although such correlation does not imply a direct functional relation, the two groups of regulatory genes would be good targets for identification of activators and repressors of *ery* expression, respectively.

### Proteomic analysis

In order to complement the results of comparative genomic and transcriptomic analysis with data on protein concentration levels we sampled the WT and HP fermentation broths at the four time points, t1– t4, and subjected the samples to quantitative proteomic analysis. In the first approach, cell lysates were separated by SDS-PAGE, analysed by LC–MS/MS, and quantified by spectral counting. 1000–1400 proteins were identified per sample at each time point. In the second approach, 2-D gel electrophoresis analysis was carried out. Reliable data for protein profiles of the samples could only be obtained for the time points t1 and t2, whereas in time points t3 and t4 extensive proteolytic degradation of the samples from the HP strain was observed in spite of using different available protease inhibitors and increasing their concentrations. Therefore, proteomic data from time points t3 and t4 were not included in data analysis. A decrease in protein abundance and in the number of detected protein spots in the later phases of the bioprocess was also confirmed by 2-D PAGE (Additional file 8).

**Fig. 4:**
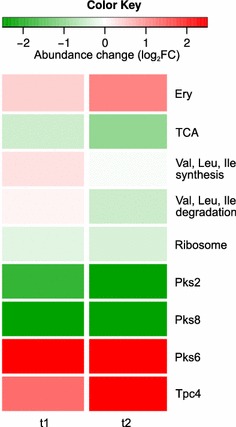
Relative changes in protein abundance (LC-MS/MS) between HP and WT strains for two time points. The changes in abundance on the level of biological processes are presented as log_2_ of the ratio between HP and WT with *red fields* representing increased abundance in HP and *green fields* representing decreased abundance in HP. Proteins are grouped according to their functional annotations in KEGG and abundance levels of group-wise averages are shown. Ery, proteins of the *ery* cluster; TCA, enzymes of the TCA cycle. Proteins of the *ery* cluster are strongly upregulated, as are some of the enzymes responsible for the synthesis of aliphatic amino acids. Enzymes of the TCA cycle are downregulated. Proteins of the *pks6* and *tpc4* secondary metabolic clusters are upregulated in HP while proteins of the *pks2* and *pks8* clusters are downregulated. Abundance changes for individual proteins belonging to each of these groups are presented in Additional file 9

The observed differences in protein abundance between the WT and HP strains (LC–MS/MS analysis) for time points t1 and t2 (Fig. 2) agree well with the results of the transcriptome analysis, with the exception that fewer proteins displayed significant changes in abundance at t2 than at t1. As shown in Fig. 4 and Additional file 9, the erythromycin-producing PKS is strongly upregulated in the HP, as are some of the enzymes involved in the branched-chain amino acid synthesis pathway. On the other hand, enzymes of the TCA cycle, ribosomal proteins, and most other proteins with significant abundance changes are downregulated in the HP strain. Some of the most striking changes in abundance were observed with proteins belonging to biosynthetic pathways of secondary metabolites: the *pks2* and *pks8* systems are strongly downregulated in the HP strain while *ery*, *pks6* and a cluster of terpenoid-quinone metabolic enzymes (*tpc4*, SACE_4645-51) are upregulated.

As a complementary method for proteome analysis we used 2-D PAGE with subsequent identification of differentially abundant proteins between HP and WT samples by LC–MS analysis (Additional file 10). This method confirmed that the protein profiles of the WT and HP strains differ significantly at time point t2. Differential abundance was observed, for example, for proteins related to glycolysis, TCA cycle, erythromycin biosynthesis, nucleotide metabolism and amino acid biosynthesis. Notably, several proteins of the carbohydrate metabolism (e.g. succinyl-CoA synthetase α subunit, SACE_6668; dihydrolipoamide succinyltransferase, SACE_1638; phosphogluconate dehydratase, SACE_1740; 6-phosphofructokinase, SACE_1704; ribose-phosphate pyrophosphokinase, SACE_0816) and nucleotide metabolism (inosine-5′-monophosphate dehydrogenase, SACE_6708; phospho-2-dehydro-3-deoxyheptonate aldolase, SACE_1708; phosphoribosylaminoimidazolecarboxamide formyltransferase, SACE_6664) were observed in the WT strain, but not in the HP strain. Additionally, more than one spot on the gel was found to belong to some of these proteins (SACE_1638, SACE_6668). Three glycolytic enzymes (glucose-6-phosphate isomerase, SACE_2158; 2-phosphoglycerate dehydratase, SACE_0838; phosphoglycerate mutase, SACE_6967) were detected only in the HP strain while glyceraldehyde 3-phosphate dehydrogenase (SACE_2143) was overexpressed in the HP strain. Additionally, the ketol-acid reductoisomerase IlvC (SACE_6157), related to valine biosynthesis, was downregulated in the HP strain. The differentially abundant proteins according to 2D PAGE are listed in Additional file 10 and are also integrated with data from other approaches in Additional file 2.

### Integrating genomics, transcriptomics and proteomics adds a new perspective

In general, when comparing data from transcriptomic and proteomic analyses (Additional file 2) we found good agreement between the expression levels of genes and abundance of the corresponding proteins. For the first two time points the observed correlation reached Pearson correlation coefficients (r) of 0.59 for t1 and 0.65 for t2 which is in the range of or even exceeding commonly reported values [26]. There are only a few exceptions where comparative transcriptomic and proteomic analyses show opposite patterns of transcript/protein abundance in WT and HP strains (Additional file 11).

**Fig. 5:**
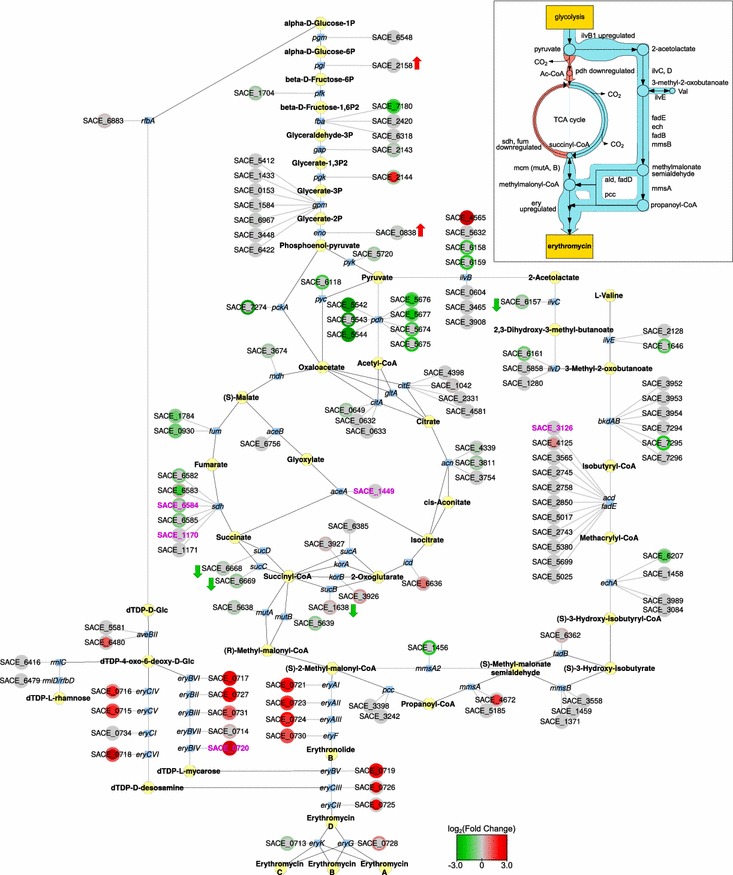
Visualisation of differences in glycolysis, TCA-cycle, valine metabolism and erythromycin biosynthesis at three omics levels. Presented data is from fermentation time point t2. Node (label) legend: *circle*: gene (locus tag), *blue rectangle*: protein (protein name), *yellow octangle*: metabolite (metabolite name). *Circle centre* and *rim colours* represent log2 (fold change between HP and WT) for gene and protein expression, respectively (see colour key). A *red arrow* next to a *circle* indicates upregulation in our 2D gel experiment, and a *green arrow* indicates downregulation. Gene mutations in the HP strain are indicated by* magenta coloured* gene labels. The inset shows a schematic representation of the proposed changes in metabolic flow between HP and WT strains. In the HP strain, key points of entry into the TCA cycle are likely restricted due to downregulation of *pdh*, *sdh*, and *fdh* (shown as *thin red lines*). Combined with the upregulation of *ilvB*, this could divert the flow of metabolites towards the biosynthesis/degradation pathway of branched-chain amino acids (shown as *thick blue lines*), resulting in increased supply of methylmalonyl-CoA and propionyl-CoA, key precursors for erythromycin synthesis

Further, we aimed to integrate diverse types of omics data to gain a more comprehensive picture of the *S.erythraea* physiology. Since the core carbon metabolism and amino acid biosynthesis exhibit significant differential expression between WT and HP, we mapped our experimental data onto these metabolic pathways to visualize their connections with erythromycin biosynthesis (Fig. 5). In the HP strain, several genes of the TCA cycle are mutated and several others are significantly downregulated compared to the WT strain. On the other hand, some genes such as *ilvB* (SACE_4565), *acd* (SACE_4125 and SACE_5025), and *mmsA* (SACE_4672) are expressed at significantly higher levels in the HP strain. Together, these data suggest that mutations and changes in the regulation of primary metabolic pathways could result in an alternative route to supplying the erythromycin precursor methylmalonyl-CoA via the branched-chain amino acid biosynthesis/degradation pathway (Fig. 5 inset). Note that several genes have multiple paralogs present in the genome (multiple gene nodes connected to the same protein node in Fig. 5) and some of these are also known to be part of secondary metabolic gene clusters. For instance, SACE_4565 (*ilvB*) and SACE_5542-4 (pyruvate dehydrogenase, *pdh*) are part of the *pks6* and *pks8* cluster, respectively.

### Re-engineering of erythromycin overproducing phenotype in the WT strain *S. erythraea*

Using a straightforward gene-overexpression approach, several genes putatively involved in increased erythromycin yield in HP strain were introduced *in trans* into the WT strain. We focused our work on three genes/operons that, based on the omics data, would most likely increase the metabolic flow towards erythromycin, when constitutively overexpressed in *S. erythraea*: (1) putative acetolactate synthase (*ilvB1*–SACE_4565) which catalyses the first dedicated step of branched-chain amino acid biosynthesis; (2) operon encoding putative branched-chain ketoacid dehydrogenase subunits (*bkdOp*–SACE_3952-54); and (3) operons encoding putative methylmalonate-semialdehyde dehydrogenase homologues, acyl-CoA dehydrogenase, enoyl-CoA hydratase and 3-hydroxybutyrate dehydrogenase (mmsOp1– SACE_1456-59 and mmsOp2– SACE_4672-73).

**Fig. 6:**
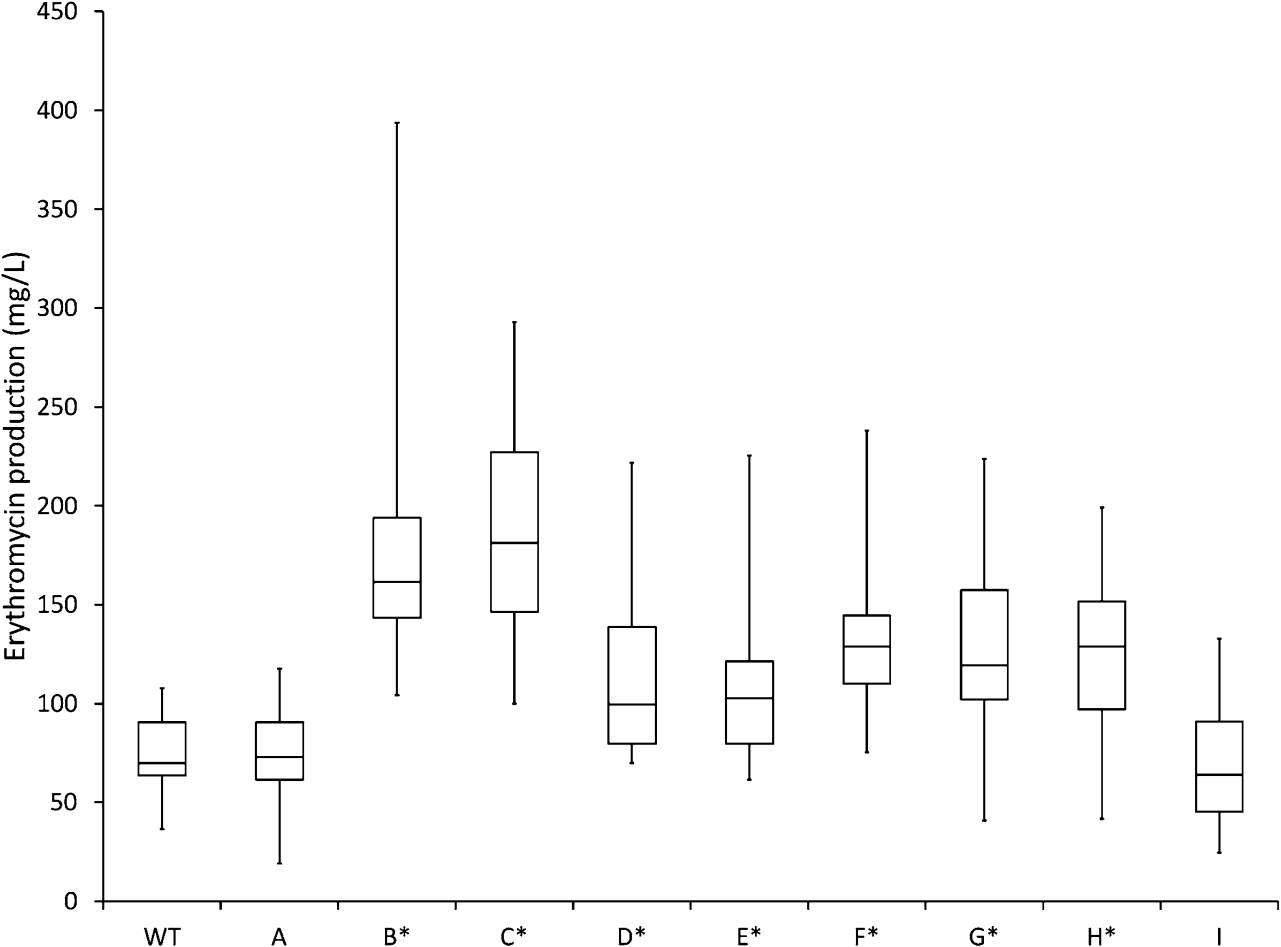
Effect of overexpressed genes on erythromycin production, compared to the WT strain. *Box plot* diagram of erythromycin production by the WT strain and transformants, determined by the microbiological assay; *WT*: control 1, NRRL2338, *A*: control 2, WT + pSet152, *B*: WT + *mmsOp1* (pABE60), *C*: WT + *mmsOp1*-HA (pABE87), *D*: WT + *ilvB1* (pABE61), *E*: WT + *ilvB1*-HA (pABE62), *F*: WT + *mmsOp1*-HA + *ilvB1*-HA (pABE89), *G*: WT + *ilvB1*-HA + *mmsOp1*-HA (pABE88), *H*: WT + *bkdOp* (pABE56), *I*: WT + *mmsOp2* (pABE95). *Boundaries of the boxes* indicate the 1st and the 3rd quartile of the sample populations. *Horizontal lines* represent the median values and *whiskers* indicate the highest and lowest values of the results. *Asterisks* denote statistically significant (p < 0.05) differences between transformants compared to control samples 1 and 2. The data were analysed using SAS/STAT program as described in the “Methods” section

All constructs were expressed under the control of the strong constitutive promoter PermE* [27]. In addition, variants of selected genes with HA-tag on the C-terminus were also expressed, to allow evaluation of expression profiles by applying western blot analysis (Additional file 12), as described previously [28]. Average erythromycin yields of all groups of transformants are presented as a box plot diagram (Fig. 6). Notably, transformants constitutively overexpressing mmsOp1 construct showed highest erythromycin yield increase (approx. 2.5 fold higher yield compared to WT strain). In addition, transformants overexpressing the *ilvB1* gene and *bkd* operon also showed significantly increased erythromycin yield, reaching 74 and 67 % erythromycin yield increase compared to the WT strain, respectively. In contrast, erythromycin yield of transformants overexpressing mmsOp2 construct did not significantly differ from the control strain. Surprisingly, when mmsOp1 operon and *ilvB1* gene were constitutively expressed together, average erythromycin yield increase of 75 % was measured, which is comparable to the yield increase achieved by *ilvB1* overexpression alone, and slightly lower compared to the transformants overexpressing only the mmsOp1 gene.

## Discussion

By applying comparative genomic, transcriptomic and proteomic analyses, we have gained a comprehensive view of the differences between WT and HP strains of *S. erythraea*. On the one hand we identified entire functional groups, e.g. metabolic pathways or biological processes (Table 1) that are expressed at different levels in the two strains. On the other hand, we used co-expression analysis to identify several possible mechanisms that could connect the genomic mutations with altered expression levels. In our analyses we assumed that mutations act directly on the affected gene, rather than through potential polar effects on downstream genes, which allowed some interpretation in terms of differences in strain physiology (Fig. 3). In general, we observed that genes/proteins related to several key physiological processes are differentially expressed in the HP strain. For example, genes related to the translation machinery (e.g. ribosomes), biosynthesis of secondary metabolites and proteolysis are significantly upregulated in HP whereas on the other hand, several key central metabolic pathways, such as the TCA-cycle and fatty acid metabolism as well as DNA repair, nucleotide metabolism and oxidative stress response are significantly downregulated. While some of these physiological features are clearly related to increased erythromycin biosynthesis, rational explanations or even just putative roles of some of the other differences observed between WT and HP strains are still to be uncovered.

In addition to the observed increased expression of dedicated erythromycin biosynthetic genes, which clearly contributes to higher yield, the supply of the main biosynthetic precursor methylmalonyl-CoA could very well be a key factor. Some genes predicted to be involved in a methylmalonyl-CoA feeder pathway are overexpressed in the HP strain, while other pathways that could contribute to a higher supply of this precursor are downregulated. It was previously demonstrated that methylmalonyl-CoA pool can either be filled or drained by the methylmalonyl-CoA mutase-catalysed reaction converting the TCA cycle intermediate succinyl-CoA and methylmalonyl-CoA. When *S. erythraea* is cultivated on carbohydrate based media this reaction proceeds in direction from methylmalonyl-CoA to succinyl-CoA, thereby consuming methylmalonyl-CoA into the TCA cycle rather than supplying precursors for erythromycin biosynthesis [29]. The observed downregulation of the TCA cycle in the HP strain may reduce this drain on the methylmalonyl-CoA pool and lead to higher erythromycin yield. In addition, our genomic and transcriptomic data show that in the HP strain the isocitrate lyase gene (SACE_1449) carries a mutation in the 5′-UTR and is significantly downregulated at the gene expression level, suggesting a possible role of conversion of methylmalonyl-CoA to succinyl-CoA as an anaplerotic step in the HP strain. Furthermore, novel findings from our transcriptomic and proteomic analyses suggest that, rather than through the TCA cycle glucose can be converted to methylmalonyl-CoA via a bypass involving genes related to branched-chain amino acid metabolism (Fig. 5). This hypothesis is also supported by the observed increased erythromycin yield in engineered strains overexpressing different genes/operons of the branched chain amino acid metabolism (Fig. 6). While branched-chain amino acids have been previously suggested as a potential source of methylmalonyl-CoA [18, 21] our results indicate that parts of biosynthesis and degradation pathways of branched-chain amino acids could operate together to provide metabolic flux to methylmalonyl-CoA (Fig. 5). Biosynthesis and degradation of e.g. valine are believed to intersect at the point of 3-methyl-2-oxobutanoate, so the metabolic flux to methylmalonyl-CoA could actually avoid valine as an intermediate (Fig. 5 inset).

In addition to the metabolic pathways involved in methylmalonyl-CoA supply, the results of our transcriptomic and proteomic studies also suggest a number of other possible mechanisms underlying increased erythromycin yield, most of which could not be validated experimentally in the scope of this study but represent a rich source of directions for future work. For example, the expression correlation analysis revealed a coordinated regulation of the *ery* cluster and several other PKS clusters (*pks2*, *pks6* and *pks8*) and a terpenoid biosynthesis cluster (*tpc4*). However, to achieve higher erythromycin titres, the HP strain has to avoid wasting resources on producing “undesired” secondary metabolites. For *pks2* and *pks8* this could be achieved by a separate mechanism of downregulation. The sigma factor SACE_5521, which appears to be connected to the expression of these two PKS clusters (Fig. 3c), would be an interesting target for further experimental characterization. The other two clusters, *pks6* and *tpc4*, are overexpressed alongside *ery*, but appear to be affected by mutations in SACE_4563 and *tauD* (SACE_4651), respectively. This could actually be advantageous to erythromycin production, as *pks6* also encodes feeder pathway proteins such as acetolactate synthase (*ilvB*-SACE_4565), which, when overexpressed, help divert substrate supply (precursors) from the primary carbon metabolism into the pool of common PKS substrate metabolites. Notably, overexpression of *pks6* cluster was also observed in other high-producing *S. erythraea* strains [18, 21]. This suggests that feeder enzymes of the SACE_4561-76 cluster continue supplying precursors, but its PKS is disabled by mutations and unable to consume the substrates, leaving more available for erythromycin biosynthesis. Thus, subverting rather than completely inactivating alternative PKS or NRPS clusters may represent one of the mechanisms for increasing the production of erythromycin or other secondary metabolites.

All experimental data generated in this study have been integrated into a spreadsheet (Additional file 2), which represents a useful tool for the identification of new targets for metabolic engineering of *S. erythraea* in the future. Notably, as the same WT strain (NRRL2338) was used as a reference in our work and in the two previous studies on independently derived erythromycin high-producing strains Px [21] and E3 [18], the available data from these studies are also included in Additional file 2 columns AN-BD. The comparative genome analysis of the strains Px [21], E3 [18] and ABE1441 (this work) shows that 60 mutations are present in all three strains (see Additional file 2), disregarding the initially excluded WT sequencing errors [19]. Out of 147 genes affected by mutations in ABE1441, 27 genes are only mutated in this high producing strain and have WT sequence in the Px and E3 strains. Each of these 27 genes harbours one variation (24 SNVs, two MNVs and one deletion; see Additional file 2). Interestingly, the uniquely mutated genes in ABE1441 include a significantly reduced expression levels of the isocitrate lyase (SACE_1449) gene, which further confirms a reduced role of the TCA cycle intermediates (e.g. succinyl-CoA) as sources for the supply of methylmalonyl-CoA extender unit in our HP strain. The high number of common mutations in the ABE1441, Px and E3 strains suggests there was a common ancestor in the strain improvement history, from which three separate development lines were subsequently initiated. In addition, several groups of genes, such as erythromycin biosynthetic genes and *pks6* (SACE_4561-76) were found to be upregulated in all three industrial strains while in contrast, the TCA cycle-related genes were downregulated.

It is important to stress that the high relevance of our results is based on a particular effort that we made to carry out omics analyses of *S. erythraea* cultures in fermentation conditions that most closely resemble conditions in the real industrial setting. Cultivation of WT and HP strains was carried out on the bioreactor scale where key process parameters such as pH and pO_2_ were controlled. Our work demonstrates that if relevant (industrial) cultivation conditions are applied and different bioinformatics and statistics approaches are used for data analysis, meaningful hypotheses can be generated and rapidly validated. Erythromycin is produced in more than 8000 tons per year [30]. To our knowledge, final yields of erythromycin at the industrial scale do not exceed 10 g/L, which is significantly less than the yields of most other mature high-volume active pharmaceutical ingredients. Considering relatively limited success in erythromycin yield improvement over decades, we believe that omics and metabolic engineering approaches based on the data presented in this work and in previously published studies [18, 19, 21] will be of particular importance for future strain and process development efforts.

## Conclusions

In conclusion, the present study contributes a significant step forward in our understanding of how erythromycin biosynthesis is coordinated with the primary carbon metabolism and with the activities of other secondary metabolic pathways of *S. erythraea* such as *pks2*, *pks6*, and *pks8*. Based on the data from omics analyses and subsequent metabolic engineering experiments we identified valine biosynthesis/degradation pathway to be of importance in providing the precursors for erythromycin biosynthesis in the HP strain. It is particularly interesting to note that in this strain an increased expression of a “competing” PKS cluster, *pks6* might further contribute to the efficiency of substrate supply for erythromycin biosynthesis. Thus, the omics data reported here suggest several future strategies for erythromycin strain improvement by metabolic engineering. Beyond that, this study demonstrates that our integrated omics approach, based on the bioprocess-guided experimental design and targeted and non-targeted data analysis, in combination with valuable literature information [18, 19, 21], generated tangible results which can be rapidly transferred to the industrial setting.

## Methods

### Genome sequencing and analysis

Since the reference WT *S. erythraea* genome was sequenced several years ago [15], we first aimed to investigate whether this reference genome assembly contains sequencing errors. We therefore analysed the recently published Illumina RNAseq reads of the same WT strain [19] [SRA accession SRX1277529], 15.7 G bases in total, and mapped them to the reference genome. Consistent variations between reference and mapped sequence reads (SNPs, insertions and deletions) were regarded as putative errors in the reference genome for all our further comparative genomic analyses. Variant analysis was performed in CLC Genomics Workbench v 6.5 (QIAGEN). Mapping to reference genome was performed using the following parameters: no masking, mismatch cost 2, insertion cost 3, deletion cost 3, length fraction 0.8, similarity fraction 0.95, global alignment ‘No’, auto-detect paired distances ‘Yes’, non-specific match handling ‘Map randomly’. CLC’s Probabilistic Variant Detection tool was used to determine potential sequencing errors of the reference genome. To ensure high confidence of variance detection, non-specific matches and broken pairs were ignored, variant presence was required in both forward and reverse reads, minimum coverage at variation sites was set to 10, variant probability to 90 % and maximum expected variants to 2.

The erythromycin high-producing ABE1441 industrial strain (HP) has been developed by Acies Bio Ltd based on the acquired intermediate producing strain. The genome of ABE1441 was sequenced using 454 sequencing technology (Roche) at Macrogen Inc., South Korea. Genomic reads were aligned to the reference genome in CLC Genomics Workbench (detailed read mapping metrics are available in Additional file 13) and CLC’s Probabilistic Variant Detection was used to determine variations. Variations that were identical to those found by aligning RNAseq reads described above [19] were flagged as WT genome sequencing errors. Some of the variations flagged as sequencing errors as well as several true HP variations were confirmed by PCR cloning and Sanger sequencing of fragments in WT and HP strains.

### Cultivation of strains and sample collection

Propagation of the *S. erythraea* WT strain and HP strain as well as genetically engineered strains was done on ABSM4 agar plates (1 % corn starch, 1.1 % corn steep liquor, 0.3 % (NH4)_2_SO_4_, 0.3 % NaCl, 0.3 % CaCO_3_, 2 % agar) for 2 weeks at 30 °C. Laboratory scale fermentation in liquid culture for estimation of erythromycin productivity of transformant strains was done in 50-ml scale. Seed cultures of all strains were prepared in the ABVM1 medium (3 % corn steep liquor, 3 % sucrose, 0.4 % (NH_4_)_2_SO_4_, 0.6 % CaCO_3_) at 30 °C and 220 rpm for 48 h.

Cultivation experiments for comparative transcriptomic and proteomic analyses were carried out in 5 L bioreactors (Sartorius Biostat B) with 3.5 L of ABPM8 production medium, operated at 30 °C, 1 vvm airflow and 350–900 rpm agitation. Bioreactors were inoculated with 10 vol. % seed culture. Dissolved oxygen was maintained above 20 % with increasing agitation and aeration rate during the bioprocess. Foaming was controlled by automatic addition of antifoam SAG5693 (Momentive). Samples from bioreactor were taken out regularly and the following parameters were determined (packed mycelium volume (PMV-%), pH, glucose concentration, erythromycin concentration). Samples for qPCR, microarray and proteomic analysis were taken during the bioprocess and stored at −80 °C until analysis as described below.

To evaluate erythromycin yield of genetically engineered strains production phase was carried out in the ABPM8 medium (3.6 % soybean flour, 3.6 % corn starch, 0.24 % (NH_4_)_2_SO_4_, 0.72 % CaCO_3_, 0.5 % soybean oil) inoculated with 10 % (v/v) of the seed culture. We have now updated the Methods section with the following sentence. “Cultivations were performed in 50 mL Falcon tubes (sealed with foam plugs) at 30 °C and 220 rpm for 7 days. The working volume was 5 mL. 2 % glucose and 0.67 % n-propanol were added at the time of inoculation. and 1 % glucose and 0.34 % n-propanol were added after 24 h of cultivation. Apramycin (50 µg/ml) and thiostrepton (25 µg/ml solid and 5 µg/ml liquid media) were added to the solid and liquid media as required.

Sampling points for transcriptomic and proteomic analysis were selected according to bioprocess parameters and marker gene expression, taking into account that different fermentations have different dynamics, as described in more detail in the “Results” section.

### Transcriptomics

Nucleotide sequences of the coding regions of *S. erythraea* were obtained from the *Saccharopolyspora erythraea* Genome Project Web Site (http://jblseqdat.bioc.cam.ac.uk/gnmweb/files.html). Agilent custom gene expression microarrays of 8×15 k format were designed using Agilent e-array (https://earray.chem.agilent.com/earray/). Positions of the predicted mutations were excluded from probe design. 60-mer probes were designed using T_m_ matching methodology (T_m_ = 80 °C, trimming allowed). Single antisense probe per gene (altogether 7216 probes) was designed and printed on microarrays in two replicates.

Cultures were fixed and RNA was isolated as described previously [28]. RNA was purified using Rneasy MinElute (Qiagen) and the quality and quantity were controlled by 2100 Bioanalyzer and RNA 6000 Nano LabChip Kit (Agilent Technologies). Sample labelling and hybridization were performed at IMGM (Germany). 300 ng of total RNA was spiked using One-Color RNA Spike-In Mix (Agilent Technologies) and subjected to reverse transcription, subsequent in vitro transcription, Cy3 labelling using Full Spectrum™ MultiStart Primers for T7 IVT (System Biosciences) and One-Color Quick-Amp Labelling Kit (Agilent Technologies) following manufacturer’s protocols. cRNA yield and integrity were determined using NanoDrop ND-1000 (Thermo Scientific) and 2100 Bioanalyzer (Agilent Technologies). cRNA was cleaned and hybridized to microarrays using Gene Expression Hybridization Kit (Agilent Technologies) following manufacturer’s protocols. Hybridized microarrays were washed using Gene Expression Wash Buffers (Agilent Technologies) followed by drying with acetonitrile. Fluorescent signal intensities were detected with Scan Control A.8.4.1 software (Agilent Technologies) on the Agilent DNA Microarray Scanner. Feature Extraction 10.7.1.1 software (Agilent Technologies) was used for feature extraction and quality control.

Raw microarray data was quality checked by inspecting signal and noise density plots, box plots, position images and QC spot intensities. Sample clustering was performed and two outlier WT samples were removed from analysis (one from time point t1 and another from time point t3).

Preliminary qPCR analysis and qPCR for validation of microarray results were carried out for selected marker genes on three independent fermentations of WT in HP strains. Primers and probes for 7 genes involved in primary metabolism and erythromycin production were designed as Custom TaqMan Gene Expression Assays (Life Technologies) and are shown in Additional file 12. Reverse transcription, qPCR setup and analysis were performed as described previously [28]. Preliminary qPCR analysis was performed on dense time point series (15–20 time points), where two genes, related to erythromycin biosynthesis were analysed (Additional file 3) while microarray validation analysis was performed at selected time points for all marker genes (Additional file 4).

### Proteomics

At selected time points 4 ml samples of fermentation broth were taken and cells were sonicated in the presence of 7 M urea, 2 M thiourea, 4 % (w/v) CHAPS, 65 mM DTT, and a protease inhibitor cocktail. Analysis of proteins in the cell extract was performed by 2-D electrophoresis [31] and LC–MS spectral count.

For LC–MS spectral count, the cell extract was separated on a 12 % SDS-PAGE gel and whole protein lanes were cut into eight bands. Following gel destaining, disulphide reduction and trypsin digestion, the peptides were extracted from the gel and loaded onto an LC–MS/MS system composed of a trapping column, an analytical column, and an Orbitrap LTQ Velos mass spectrometer (Thermo Scientific). MS/MS spectra were obtained by fragmentation of the nine most intense precursor ions from the full MS scan. The database search and quantification by spectral counting were performed using the MaxQuant proteomics software [32, 33] using the *S. erythraea* protein database obtained from the *Saccharopolyspora erythraea* Genome Project Web Site.

2-D electrophoresis was run on IPG strips (pH 4–7) for the first dimension and a 12 % SDS-PAGE gel for the second dimension, then gels were stained with SYPRO Ruby (Invitrogen). Triplicate gels for each sample were matched to provide an average gel sample. Full experimental details of proteomic analysis are provided in Additional file 13.

### Data analysis

Differential gene expression analysis was performed in the R statistical environment using limma package [34]. Features with signal intensity lower than ‘the average background signal increased for 2-times standard deviation’ in less than three samples were excluded from further analysis. Raw data were log-transformed, quantile-normalized and averaged between the two replicated probes. Log fold changes (logFC) for comparison between HP and WT strain in each time point were calculated. Statistics for the comparisons were calculated using the eBayes function. Genes having *p* value <0.01 and |logFC| > 1.0 were considered to be significantly differentially expressed. Additionally, gene expression profiles of the two strains were analysed using regression based approach implemented in the maSigPro Bioconductor package [35].

Enrichment analysis was performed using normalized averaged signals for each time point separately in GSEA desktop application v2.2.2 using “gene_set” permutation type, “Signal2Noise” method for ranking genes and *S. erythraea* KEGG BRITE or GO ontology (generated by Blast2Go software, BioBam) to build gene sets. KEGG gene sets with less than 8 or more than 400 and GO gene sets with less than 15 and more than 500 genes were excluded from analysis. Gene sets with enrichment nominal p value <0.01 for KEGG and p < 0.05 for GO were considered to be statistically significant.

For LC–MS spectral counts protein abundance ratio between HP and WT strain (logFC) of the same samples as used for transcriptomics was calculated for time points t1 and t2. LogFC was calculated for proteins with at least one spectral count above 10. Proteins with |logFC| > 1.5 were considered to be differentially abundant. LogFC values for time points t3 and t4 were not calculated because of the extensive proteolytic degradation of the samples from the HP strain in these time points. Spots on 2-D electrophoresis gels were visualized and quantified using the 2-D Dymension software, version 2.02 (Syngene), on the basis of their normalized volumes, defined as the spot volume divided by the total volume over the whole set of gel spots. Expression changes (HP/WT) for particular time point were considered as significant when the intensity of the corresponding spots reproducibly differed by more than 1.5-fold in a normalized volume (p value <0.05). The differentially expressed proteins were cut from the gel and identified by mass spectrometry as describe in detail in Additional file 13.

Analysis of correlation coefficients was carried out using a custom-written R script. Three separate matrices of Pearson’s correlation coefficients were calculated, one including all experimental data simultaneously, a second using only data from the WT strain, and a third using only data from the HP strain. To calculate p-values for correlation coefficients in each matrix, we randomly reshuffled the order of all measurements corresponding to each gene and recalculated the correlation matrix using the reshuffled data, which yielded random correlation coefficients without changing the distribution of expression levels for each gene. The distribution of these randomized correlation coefficients was then used to calculate the probability of a given value of a correlation coefficient appearing at random (p value). We also calculated the FDR q values. The calculation of correlation coefficients and q values, as well as the random reshuffling of experimental data was accomplished using built-in functions in R.

For each gene, we calculated the sum of squared correlation coefficients to all members of the *ery* cluster and the maximum squared correlation coefficient to any gene harbouring a mutation in the HP strain. These values, calculated using overall as well as WT- and HP-only correlations, were used to filter genes. Typically only the top 5 % of genes according to each criterion were considered. The correlations between selected genes were visualized in Cytoscape and filtered to produce visually interpretable figures. This typically required very stringent criteria with p values for individual correlations <0.005.

Genomic variation, microarray, qPCR, 2-D PAGE and shotgun proteomics results generated in this study were additionally integrated with gene annotations from several sources and gene expression/variation results from three recently published transcriptomic studies [19–21]. Comprehensive presentation of all data from our genomic, transcriptomic and proteomic experiments as well as data from relevant previous studies is presented in Additional file 2.

### Overexpression of selected genes in *S. erythraea*

To confirm that the observed differences between WT and HP strains indeed result in increased erythromycin yield, we overexpressed several genes, related to branched-chain amino acid metabolism in the WT strain. Selected genes/putative operons were amplified using *S. erythraea* genomic DNA as template. Sequences of primers used for amplification of target DNA segments are presented in Additional file 12. In all cases NdeI restriction site was introduced at the 5′-end and XbaI site was inserted at the 3′-end, after the stop codon. The PCR amplified fragments were cloned into the pSet152-derived plasmid into which the constitutive PermE* promoter [27] had been previously cloned, thus creating a series of pABE vectors (Additional file 14). Correct assembly of the PCR products was confirmed by sequencing. In the cases of *ilvB* gene and mmsOp constructs additional variants with a HA-tag on the C-terminus of the proteins were prepared in order to enable confirmation of functional expression of overexpressed proteins by western blotting.

Simultaneous overexpression of *ilvB1* gene and *mms* operon genes was also carried out. For this purpose two pSet152 based vectors were constructed (pABE88 and pABE89) in which the *ilvB1* gene and *mms* operon were positioned in different orders. In pABE89 *ilvB1* gene was cloned upstream of the *mms* operon genes, whereas in pABE88 construct *ilvB1* was positioned downstream of the *mms* operon. In both cases, expression of the upstream gene was driven directly by the PermE* promoter, while for expression of the downstream gene a RBS was introduced upstream of the gene’s start codon. The obtained plasmid constructs were introduced into *S. erythraea* WT strain using standard conjugation procedure [36]. The obtained independent colonies were cultivated in shake flasks and erythromycin yields were estimated by microbiological assay and HPLC analyses according to previously described procedures [28].

For testing the effect of different mutations on erythromycin production, compared to the WT strain, at least 20 independent colonies (transformants) were tested for each engineered strain in two consecutive independent experiments. Each independent colony (transformant) was tested in duplicates. Yields of erythromycin were calculated with SAS/STAT software using means and the univariante procedure to test the normality of distribution. Using the GLM model, data were calculated as least mean square and are presented as an average change observed from all experiments when comparing least mean square values to the wild-type control least mean square value of each experiment.

## Additional files

10.1186/s12934-016-0496-5 40 putative differences between the WT DNA sequence and published RNA-seq data [SRA Accession SRP014619] that were considered to be sequencing errors of the original genome sequence.
10.1186/s12934-016-0496-5 An integrated table of all obtained genomic, transcriptomic and proteomic data as well as data obtained and published in previous studies (Peano et al., 2012; Marcellin et al., 2013, Li et al., 2011).
10.1186/s12934-016-0496-5 A graphic representation of erythromycin biosynthesis genes *eryK* and *eryAI* in 6 individual fermentations of wild type and high producer strain of *S. erythraea*, analysed by qPCR.
10.1186/s12934-016-0496-5 A table for validation of microarray data by qPCR.
10.1186/s12934-016-0496-5 A table of overrepresentation of GO Molecular function gene sets (KEGG pathways or GO Biological processes) in *S. erythraea* HP compared to WT strain in individual time points (t1-t4).
10.1186/s12934-016-0496-5 A graphical representation of clusters of genes with differential time-course profiles between the HP and WT strains.
10.1186/s12934-016-0496-5 A graphic representation of regulatory genes whose expression profiles exhibit strong correlations to *ery* genes.
10.1186/s12934-016-0496-5 Results of the 2-D PAGE experiments: representation of 2-D gels of WT and HP strain in different time points (t1-t4), comparison of 2-D images between HP and WT in t1 and t2.
10.1186/s12934-016-0496-5 A heatmap representation of LC–MS proteomics results on individual proteins belonging to selected metabolic pathways.
10.1186/s12934-016-0496-5 A table of differentially expressed proteins according to 2-D PAGE: KEGG Pathway classification of differentially expressed proteins in time points 1 and 2.
10.1186/s12934-016-0496-5 A table of genes and proteins with apparently opposite regulation patterns in transcriptomic and high-throughput proteomic profiling.
10.1186/s12934-016-0496-5 Figures representing western blot analyses of constitutively over-expressed *ilvB1* gene and *mms* operon (*mmsOp*).
10.1186/s12934-016-0496-5 A detailed description of selected methods: preliminary qPCR analysis of key erythromycin related genes, ABE1441 strain sequencing and read mapping metrics, proteomic analysis (2-D electrophoresis, spectral counting), oligonucleotide primers used in genetic engineering of *S. erythraea.*

10.1186/s12934-016-0496-5 A table with description of strains and plasmids generated and used in this study and graphical representation of over-expressed operons/genes.

## Abbreviations

2-D: two dimensional
CHAPS: 3-[(3-cholamidopropyl)dimethylammonio]-1- propanesulfonate
CoA: coenzyme A
DE: differentially expressed
DTT: dithiothreitol GSEA: gene set enrichment analysis
HA: human influenza hemagglutinin
HP: *S. erythraea* high-producing strain ABBE1441
LC–MS: liquid chromatography–mass spectrometry
MNV: multiple nucleotide variation
NRPS: non-ribosomal peptide synthetase
ORF: open reading frame
TCA: tricarboxylic acid
PAGE: polyacrylamide gel electrophoresis
PCR: polymerase chain reaction
PMV: packed mycelium volume
PKS: polyketide synthase
qPCR: quantitative real-time PCR
RBS: ribosomal binding site
SDS: sodium dodecyl sulphate
rpm: revolutions per minute
SNV: single nucleotide variation
UTR: untranslated region
vvm: volume of air under standard conditions per volume of liquid per minute
WT: *S. erythraea* wild type strain NRRL2338
