# Supplementary material for: Integrated omics approaches provide strategies for rapid erythromycin yield increase in Saccharopolyspora erythraea
Source: Microb Cell Fact. 2016 Jun 3;15:93. doi: 10.1186/s12934-016-0496-5 (PMC4891893; doi:10.1186/s12934-016-0496-5)
Supplement: Supplementary file 3 — 10.1186/s12934-016-0496-5 A graphic representation of erythromycin biosynthesis genes eryK and eryAI in 6 individual fermentations of wild type and high producer strain of S. erythraea, analysed by qPCR. [file 12934_2016_496_MOESM3_ESM.pdf]

Additional file 3: a) Relative gene expression of erythromycin biosynthesis genes *eryK* and *eryAI* (normalized to the expression of 16S rRNA; arbitrary units) in 6 individual fermentations of wild type (WT1-3, left) and high producer (HP1-3, right) strain of *S. erythraea*, analysed by qPCR. b) Experimental time points from individual fermentations shown in a) chosen for transcriptome and proteome sampling (t1-t4) on the basis of gene expression and bioprocess parameters.

a)

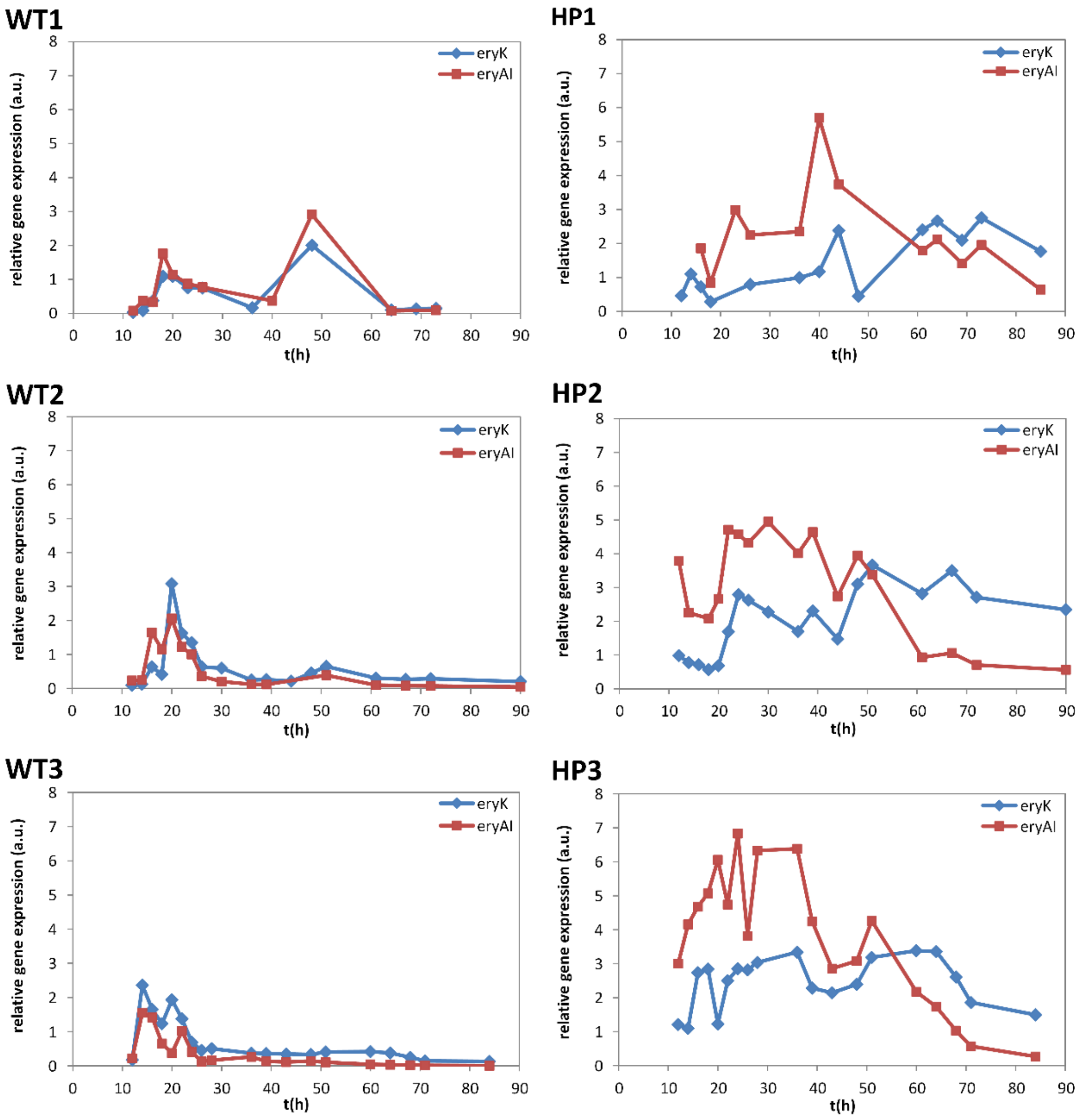

b)

fermentation/time (h)	t1	t2	t3	t4
**WT1**	18	26	48	73
**WT2**	20	26	51	72
**WT3**	20	24	51	71
**HP1**	36	40	44	85
**HP2**	24	29	51	90
**HP3**	24	26	51	84
