# Supplementary material for: Integrated omics approaches provide strategies for rapid erythromycin yield increase in Saccharopolyspora erythraea
Source: Microb Cell Fact. 2016 Jun 3;15:93. doi: 10.1186/s12934-016-0496-5 (PMC4891893; doi:10.1186/s12934-016-0496-5)

Additional file 4: Validation of microarray data using qPCR. A) Log_2 of ratios (logFC) between the expression in HP and WT *S. erythraea* strains in four time points of the fermentation (t1-t4) obtained by microarray (columns) or qPCR (diamond) analysis for 7 marker genes are shown. Statistically non-significant changes are shown in grey. B) Correlation between microarray and qPCR data for all samples/genes. Person correlation coefficient between microarray and qPCR data was 0.84. Gene descriptions and their primer sequences are shown in Additional file 14.

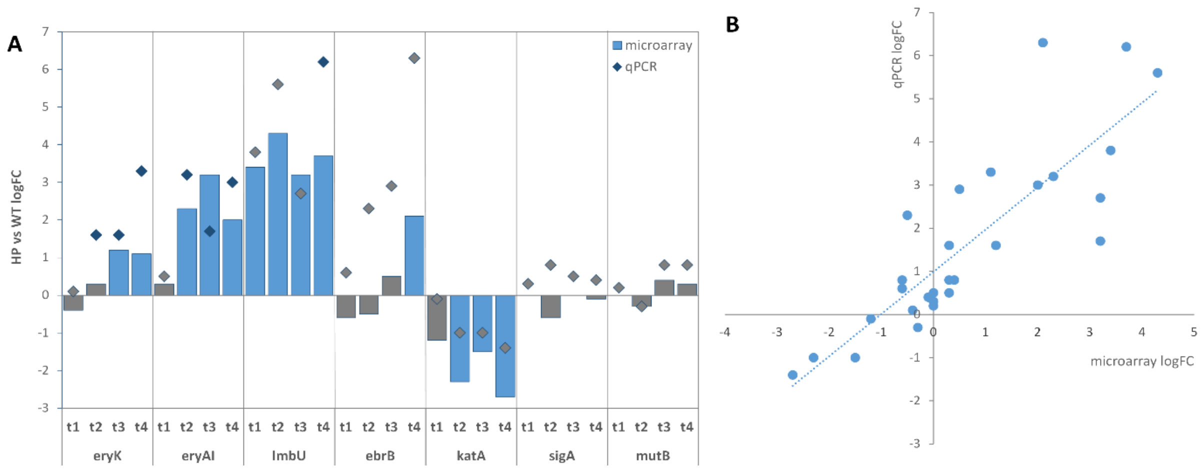

Supplement: Supplementary file 4 — 10.1186/s12934-016-0496-5 A table for validation of microarray data by qPCR. [file 12934_2016_496_MOESM4_ESM.pdf]
