# Supplementary material for: Integrated omics approaches provide strategies for rapid erythromycin yield increase in Saccharopolyspora erythraea
Source: Microb Cell Fact. 2016 Jun 3;15:93. doi: 10.1186/s12934-016-0496-5 (PMC4891893; doi:10.1186/s12934-016-0496-5)
Supplement: Supplementary file 5 — 10.1186/s12934-016-0496-5 A table of overrepresentation of GO Molecular function gene sets (KEGG pathways or GO Biological processes) in S. erythraea HP compared to WT strain in individual time points (t1-t4). [file 12934_2016_496_MOESM5_ESM.pdf]

Additional file 5

Table: Overrepresentation of GO Molecular function gene sets (KEGG pathways or GO Biological process) in *S. erythrea* HP compared to WT strain in individual time points (t1-t4). Gene set names, codes and sizes (total numbers of *S. erythrea* genes associated with the gene set) are shown. Significant enrichment (p<0.05) of the individual gene sets in up- or down-regulated genes is represented by '+' or '-', respectively.

**GO Molecular function gene sets**			**HP vs WT**			
**Name**	**Code**	**Size**	**t1**	**t2**	**t3**	**t4**
Structural constituent of ribosome	GO:0003735	59	+	+		+
rRNA binding	GO:0019843	35	+	+		+
Iron-sulfur cluster binding	GO:0051536	24	+			
Iron ion binding	GO:0005506	35				+
Heme binding	GO:0020037	49				+
Pyridoxal phosphate binding	GO:0030170	71		+	+	
Phosphopantetheine binding	GO:0031177	17	-			
Oxidoreductase activity	GO:0016491	65		-		
Oxidoreductase activity, acting on paired donors, with incorporation or reduction of molecular oxygen	GO:0016705	32				+
Ligase activity	GO:0016874	20		-		
Transporter activity	GO:0005215	84		-		
Amino acid transmembrane transporter activity	GO:0015171	27	-			
Sigma factor activity	GO:0016987	37	-	-	-	
Transposase activity	GO:0004803	53		-	-	-
