# Supplementary figures and images for: Integrated omics approaches provide strategies for rapid erythromycin yield increase in Saccharopolyspora erythraea
Source: Microb Cell Fact. 2016 Jun 3;15:93. doi: 10.1186/s12934-016-0496-5 (PMC4891893; doi:10.1186/s12934-016-0496-5)

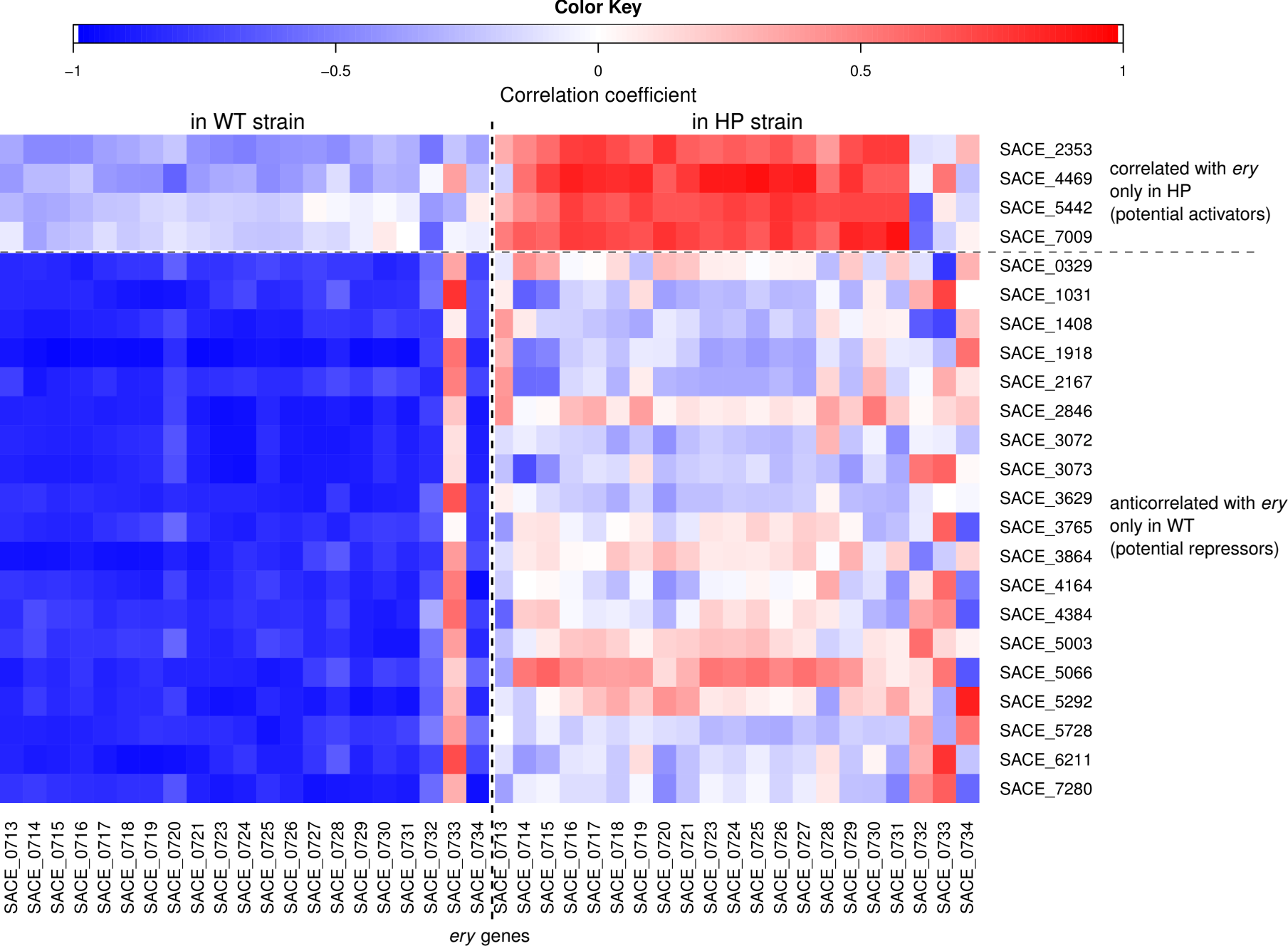

Supplement: Supplementary file 7 — 10.1186/s12934-016-0496-5 A graphic representation of regulatory genes whose expression profiles exhibit strong correlations to ery genes. [file 12934_2016_496_MOESM7_ESM.pdf]
