# Supplementary material for: Integrated omics approaches provide strategies for rapid erythromycin yield increase in Saccharopolyspora erythraea
Source: Microb Cell Fact. 2016 Jun 3;15:93. doi: 10.1186/s12934-016-0496-5 (PMC4891893; doi:10.1186/s12934-016-0496-5)
Supplement: Supplementary file 8 — 10.1186/s12934-016-0496-5 Results of the 2-D PAGE experiments: representation of 2-D gels of WT and HP strain in different time points (t1-t4), comparison of 2-D images between HP and WT in t1 and t2. [file 12934_2016_496_MOESM8_ESM.pdf]

Additional file 8. 2-D gel analysis

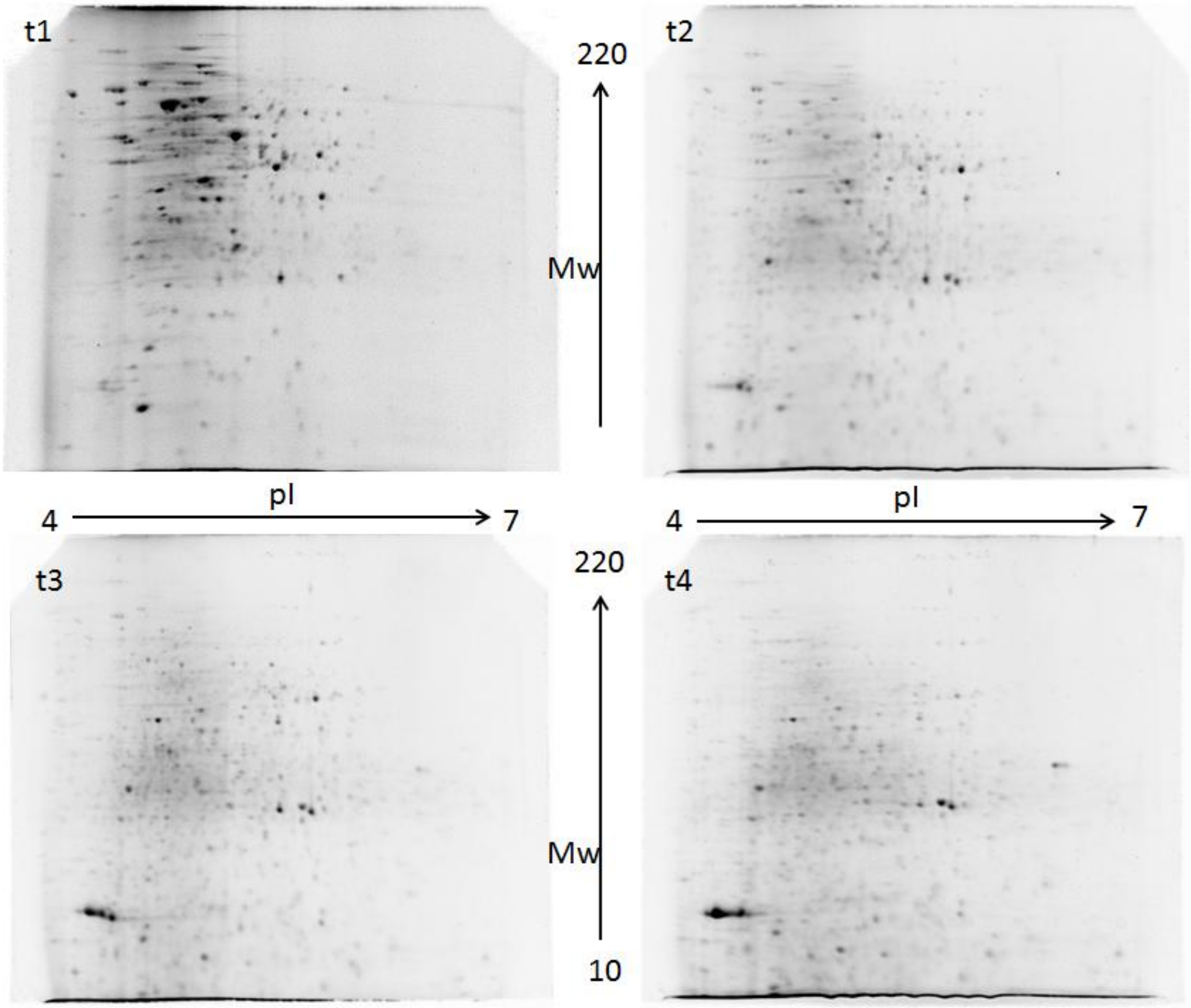

Figure A: Representation of 2-D gels of WT strain in different time points (t1-t4)

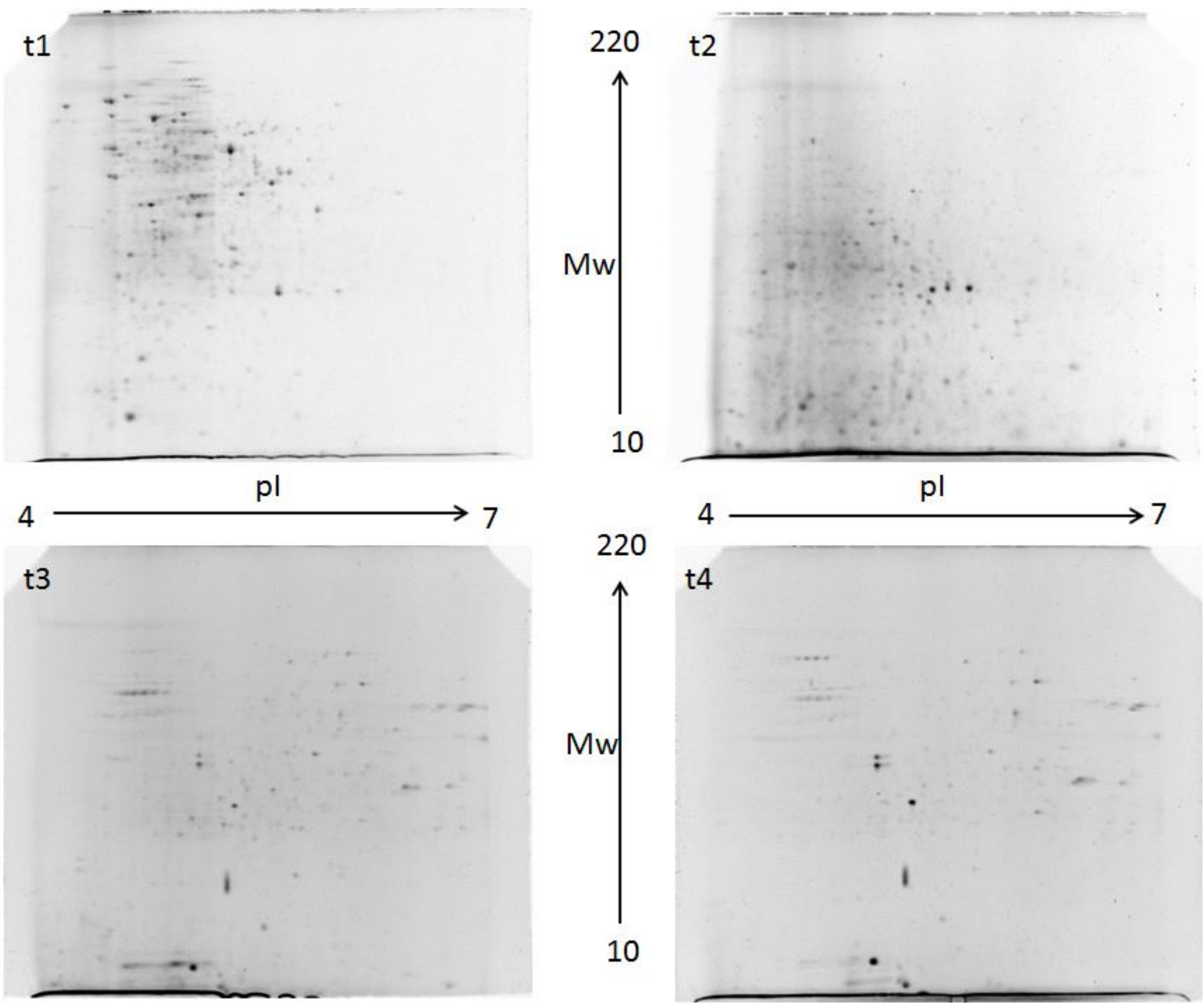

Figure B: Representation of 2-D gels of HP strain in different time points (t1-t4)

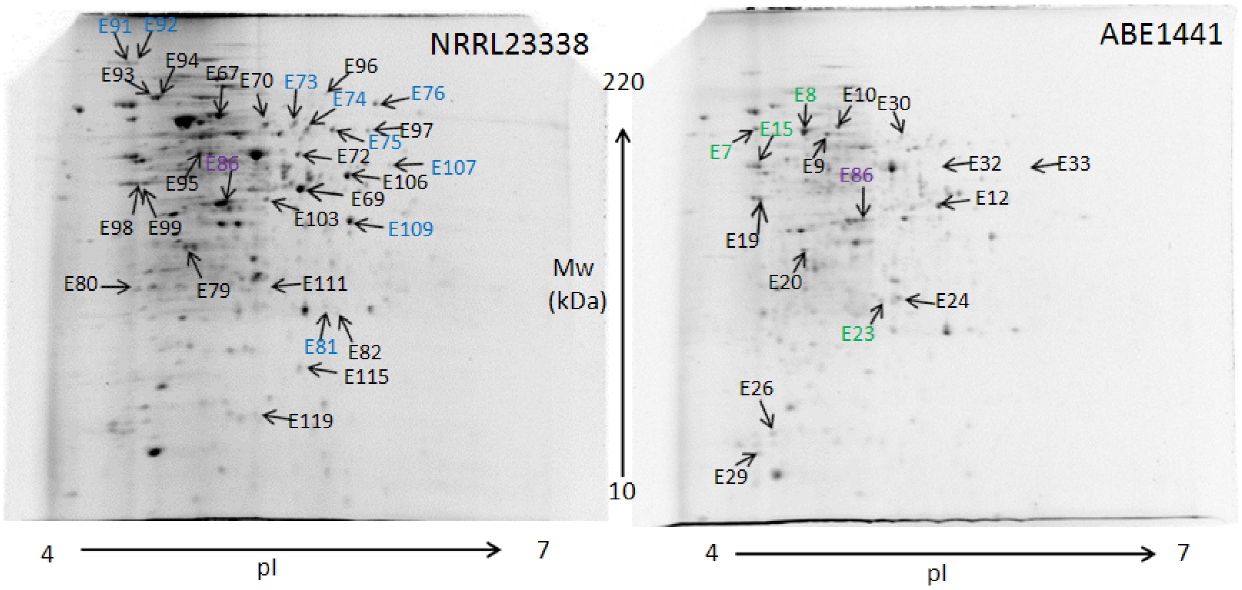

Figure C: Comparison of 2-D images between HP and WT in t1 (see spot identification in table 1). Spots marked with blue color mark proteins observed in the WT strain, but not in the HP strain (SACE_6668, SACE_1638, SACE_1740, SACE_1704, SACE_0816, SACE_6708, SACE_1708, SACE_6664); spots marked with green color mark proteins observed only in the HP strain, but not in WT strain; spot marked with purple marks ketol-acid reductoisomerase IlvC (SACE_6157) that was downregulated in the HP strain.

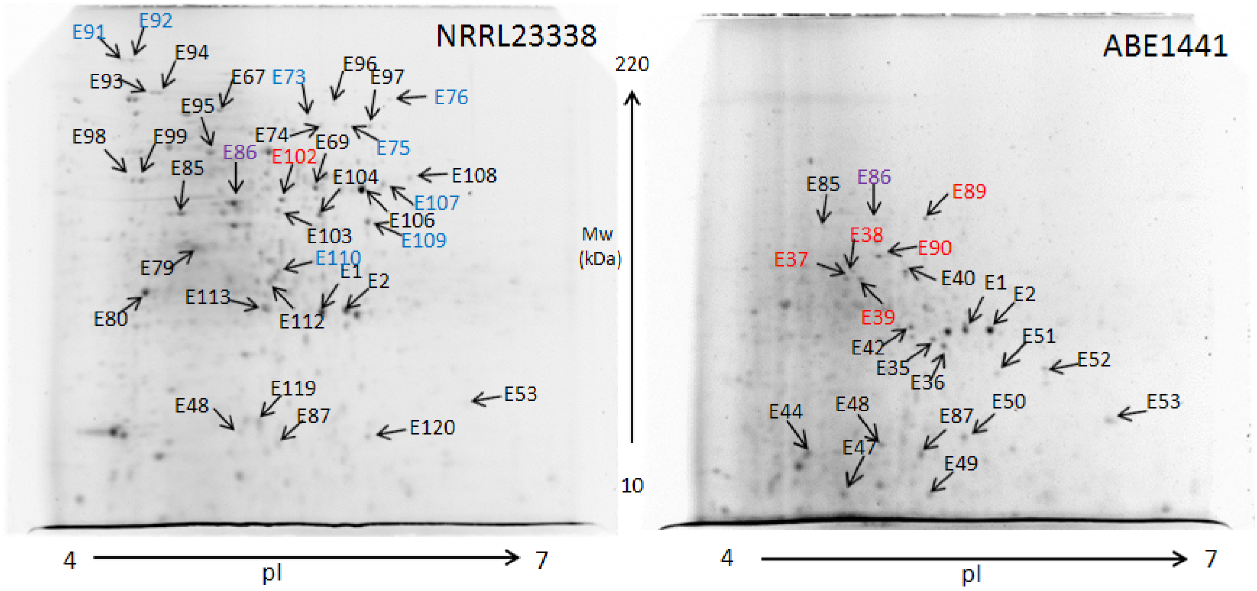

Figure D: Comparison of 2-D images between HP and WT in t2 (see spot identification in table below). Spots marked with blue color mark proteins observed in the WT strain, but not in the HP strain (SACE_6668, SACE_1638, SACE_1740, SACE_1704, SACE_0816, SACE_6708, SACE_1708, SACE_6664); spots marked with red color mark erythromycin 3''-O-methyltransferase EryG (SACE_0728) that was found at five different locations in the HP strain but only in one spot in the WT strain; spot marked with purple marks ketol-acid reductoisomerase IlvC (SACE_6157) that was downregulated in the HP strain.

Table: Spot identification

Spot no.	Protein
E1	superoxide dismutase [Fe-Zn] 1 (SACE_0619)
E2	putative glycosyltransferase (SACE_2010)
E7, E8	glucose-6-phosphate isomerase (SACE_2158)
E9, E10, E51	D-3-phosphoglycerate dehydrogenase (SACE_6155)
E12, E40, E52, E69, E103	glyceraldehyde 3-phosphate dehydrogenase (SACE_2143)
E15	enolase/2-phospho-d-glycerate hydro-lyase/2-phosphoglycerate dehydratase (SACE_0838)
E19, E98, E99	succinyl-CoA ligase [ADP-forming] subunit (SACE_6669)
E20	electron transfer flavoprotein subunit alpha (SACE_6196)
E23	phosphoglycerate mutase (SACE_6967)
E24, E111	ATP synthase subunit delta/ATP synthase F(1) sector subunit delta (SACE_6283)
E26	putative fructose-specific permease (SACE_2274)
E29	transcription elongation factor GreA/transcript cleavage factor GreA (SACE_0907)
E30, E70	dihydrolipoamide dehydrogenase (SACE_5677)
E32	isocitrate lyase (SACE_1449)
E33	putative L-alanine dehydrogenase (SACE_6380)
E35, E36	malate dehydrogenase (SACE_3674)
E37, E38, E39, E89, E99, E102	erythromycin 3''-O-methyltransferase (SACE_0728)
E42, E85, E113	thiosulfate sulfurtransferase (SACE_7106)
E44	NAD-dependent epimerase/dehydratase family protein (SACE_0813)
E47	Elongation factor Ts (EF-Ts) (SACE_6037)
E48	heat shock protein HSP20 (SACE_0150)
E49	ATP synthase subunit alpha/ATP synthase F1 sector subunit alpha (SACE_6282)
E50	DNA-directed RNA polymerase subunit beta/RNAP subunit beta (SACE_6853)
E53	Acetyltransferase (SACE_5342)
E67	Arginine--tRNA ligase/Arginyl-tRNA synthetase (SACE_6300)
E72	isocitrate lyase (SACE_1449)
E73	inosine-5'-monophosphate dehydrogenase (SACE_6708)
E74	phospho-2-dehydro-3-deoxyheptonate aldolase (SACE_1708)
E75	phosphoribosylaminoimidazolecarboxamide formyltransferase/IMP cyclohydrolase (SACE_6664)
E76	phosphogluconate dehydratase (SACE_1740)
E79, E80, E119	ATP synthase beta chain (SACE_6280)
E81	ribose-phosphate pyrophosphokinase (SACE_0816)
E82	flavodoxin/nitric oxide synthase (SACE_2881)
E86	ketol-acid reductoisomerase/acetohydroxy-acid isomeroreductase (SACE_6157)
E87	probable fatty acid oxidation complex alpha subunit (SACE_1823)
E91, E92	dihydrolipoamide succinyltransferase (SACE_1638)
E93, E94, E95	70 kD heat shock protein (molecular chaperone) (SACE_7210)
E96	delta-1-pyrroline-5-carboxylate dehydrogenase precursor (SACE_1979)
E97	phosphodiesterase/alkaline phosphatase D (SACE_1400)
E104, E106, E120	oxidoreductase (SACE_0698)
E107	6-phosphofructokinase (SACE_1704)
E108	4-hydroxyphenylpyruvate dioxygenase (SACE_0905)
E109, E110	succinyl-CoA synthetase alpha chain (SACE_6668)
E112	phosphate transport system regulator PhoU-related protein (SACE_7091)
E115	plant-type carbonic anhydrase (SACE_0433)
