# Supplementary material for: Integrated omics approaches provide strategies for rapid erythromycin yield increase in Saccharopolyspora erythraea
Source: Microb Cell Fact. 2016 Jun 3;15:93. doi: 10.1186/s12934-016-0496-5 (PMC4891893; doi:10.1186/s12934-016-0496-5)
Supplement: Supplementary file 9 — 10.1186/s12934-016-0496-5 A heatmap representation of LC–MS proteomics results on individual proteins belonging to selected metabolic pathways. [file 12934_2016_496_MOESM9_ESM.pdf]

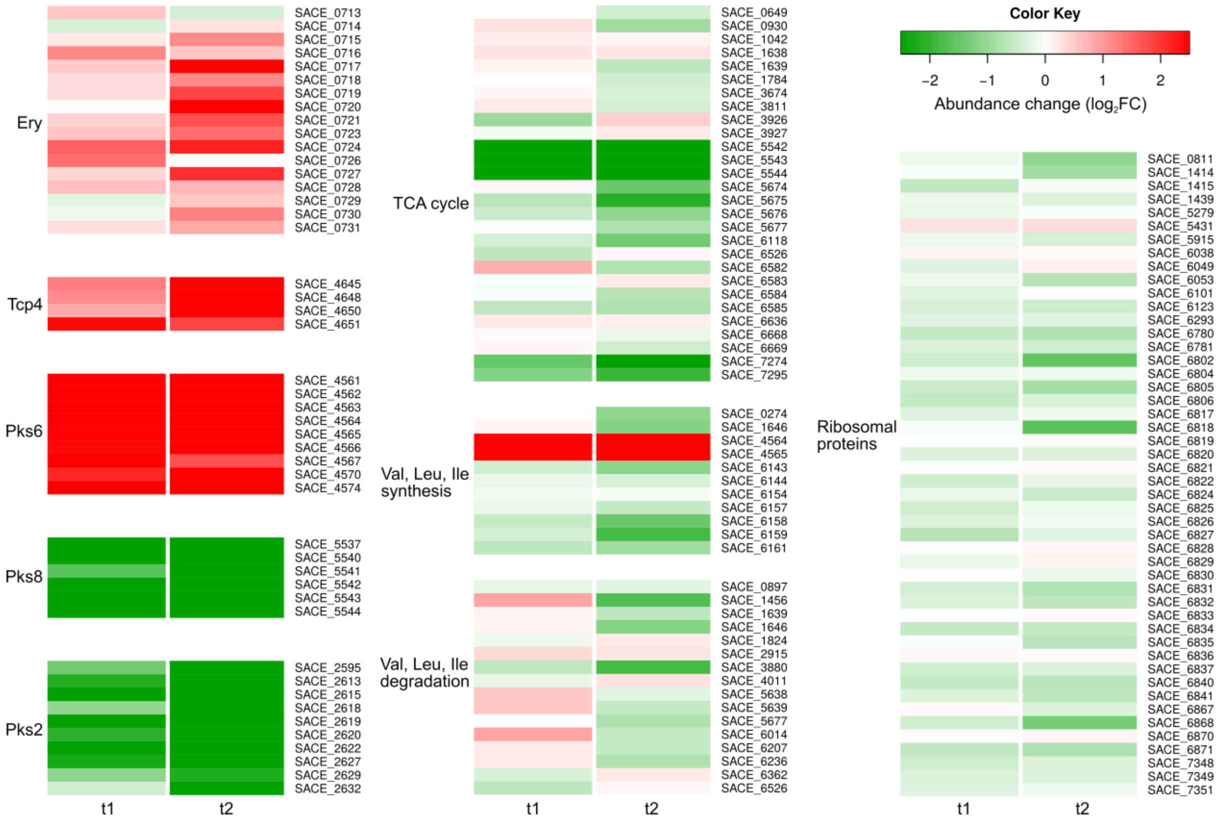

Additional file 9: Heatmap plot of the relative changes in protein expression between HP and WT strains for proteins in select functional groups at two different timepoints. The changes in expression are presented as $\log_2$ of the ratio between raw expression in HP and expression in WT with red fields representing upregulation in HP and green fields representing downregulation in HP. Proteins are labeled according to their gene names and split into functional groups according to KEGG pathway annotations.
