# Supplementary material for: Integrated omics approaches provide strategies for rapid erythromycin yield increase in Saccharopolyspora erythraea
Source: Microb Cell Fact. 2016 Jun 3;15:93. doi: 10.1186/s12934-016-0496-5 (PMC4891893; doi:10.1186/s12934-016-0496-5)
Supplement: Supplementary file 10 — 10.1186/s12934-016-0496-5 A table of differentially expressed proteins according to 2-D PAGE: KEGG Pathway classification of differentially expressed proteins in time points 1 and 2. [file 12934_2016_496_MOESM10_ESM.pdf]

Additional file 10: Table of differentially expressed proteins according to 2-DE (KEGG Pathway classification of differentially expressed proteins in time points 1 and 2)

KEGG classification	Down regulation		Up regulation	
Carbohydrate & energy metabolism	dihydrolipoamide dehydrogenase (SACE_5677)	t1	succinyl-coa ligase [adp-forming] subunit beta/succinyl-coa synthetase subunit beta (SACE_6669)	t1
	glyceraldehyde 3-phosphate dehydrogenase (SACE_2143)	t1	glucose-6-phosphate isomerase (SACE_2158)	t1, t2
	isocitrate lyase (SACE_1449)	t1	enolase/2-phospho-d-glycerate hydro-lyase/2-phosphoglycerate dehydratase (SACE_0838)	t1, t2
	ATP synthase subunit delta/atp synthase f(1) sector subunit delta (SACE_6283)	t1	phosphoglycerate mutase (SACE_6967)	t1
	phosphogluconate dehydratase (SACE_1740)	t1, t2	putative fructose-specific permease (SACE_2274)	t1
	ATP synthase beta chain (SACE_6280)	t1, t2	D-3-phosphoglycerate dehydrogenase (SACE_6155)	t1, t2
	ribose-phosphate pyrophosphokinase (SACE_0816)	t1, t2	glyceraldehyde 3-phosphate dehydrogenase (SACE_2143)	t2
	dihydrolipoamide succinyltransferase (SACE_1638)	t1, t2	malate dehydrogenase (SACE_3674)	t2
	6-phosphofructokinase (SACE_1704)	t1, t2	ATP synthase subunit alpha/ATP synthase F1 sector subunit alpha (SACE_6282)	t2
	succinyl-coa synthetase alpha chain (SACE_6668)	t1, t2		
	plant-type carbonic anhydrase (SACE_0433)	t1, t2		
	thiosulfate sulfurtransferase (SACE_7106)	t2		
Lipid metabolism			probable fatty acid oxidation complex alpha subunit (SACE_1823)	t2
Nucleotide metabolism	inosine-5'-monophosphate dehydrogenase (SACE_6708)	t1, t2	DNA-directed rna polymerase subunit beta/rnap subunit beta (SACE_6853)	t2
	phospho-2-dehydro-3-deoxyheptonate aldolase (SACE_1708)	t1, t2		
	phosphoribosylaminoimidazolecarboxamide formyltransferase/imp cyclohydrolase (SACE_6664)	t1, t2		
Metabolism of terpenoids and polyketides			erythromycin 3''-o-methyltransferase (SACE_0728)	t2
Amino acid	ketol-acid reductoisomerase/acetohydroxy-acid	t2	putative L-alanine dehydrogenase (SACE_6380)	t1

metabolism	isomeroreductase (SACE_6157)			
	delta-1-pyrroline-5-carboxylate dehydrogenase precursor (SACE_1979)	t1, t2		
	4-hydroxyphenylpyruvate dioxygenase (SACE_0905)	t1, t2		
Xenobiotics biodegradation and metabolism	phosphodiesterase/alkaline phosphatase D (SACE_1400)	t1, t2		
Translation	arginine--tRNA ligase/arginyl-tRNA synthetase (SACE_6300)	t1, t2	elongation factor Ts (EF-Ts) (SACE_6037)	t2
Folding, sorting and degradation	heat shock protein HSP20 (SACE_0150)	t2		
	70 kd heat shock protein (molecular chaperone) (SACE_7210)	t1, t2		
Other	superoxide dismutase [Fe-Zn] 1 (SACE_0619)	t2	putative glycosyltransferase (SACE_2010)	t2
	acetyltransferase (SACE_5342)	t2	electron transfer flavoprotein, alpha subunit (SACE_6196)	t1
	flavodoxin/nitric oxide synthase (SACE_2881)	t1, t2	transcription elongation factor GreA/transcript cleavage factor GreA (SACE_0907)	t1
	oxidoreductase (SACE_0698)	t1, t2	NAD-dependent epimerase/dehydratase family protein (SACE_0813)	t2
	phosphate transport system regulator PhoU-related protein (SACE_7091)	t1, t2		

t1, t2 – differentially expressed in timepoint 1 (t1) or timepoint 2 (t2)
