# Supplementary material for: Integrated omics approaches provide strategies for rapid erythromycin yield increase in Saccharopolyspora erythraea
Source: Microb Cell Fact. 2016 Jun 3;15:93. doi: 10.1186/s12934-016-0496-5 (PMC4891893; doi:10.1186/s12934-016-0496-5)
Supplement: Supplementary file 11 — 10.1186/s12934-016-0496-5 A table of genes and proteins with apparently opposite regulation patterns in transcriptomic and high-throughput proteomic profiling. [file 12934_2016_496_MOESM11_ESM.pdf]

Additional file 11

Table: Genes and proteins with apparently opposite regulation patterns in transcriptomic and high-throughput proteomic profiling.

		microarray		proteomics	
**Locus ID**	**Description**	**t1 logFC**	**t2 logFC**	**t1 logFC**	**t2 logFC**
SACE_1764	ribonucleoside diphosphate reductase	1.82		-1.42	
SACE_2159	tal - transaldolase	1.44	1.51	-1.53	-0.01
SACE_5340	6-phosphogluconate dehydrogenase	1.52	1.15	-1.24	-1.2
SACE_5814	cypA - cytochrome P450-like enzyme	1.31	1.03	-1.11	0.43
SACE_6196	fixB - electron transfer flavoprotein	1.52	0.99	-1.15	-0.02
SACE_6298	thrA - homoserine dehydrogenase	1.17		-1.14	
SACE_6447	mtrA - two-component response regulator	1.04	0.67	-3.2	-2.06
SACE_6802	rplQ - 50S ribosomal protein L17	1.4	1.3	-1.4	-0.6
SACE_6868	rplJ - 50S ribosomal protein L10	1.91	3.01	-1.2	-0.55
SACE_7385	trxB - thioredoxin reductase	1.22		-1.45	
SACE_6380	ald - putative L-alanine dehydrogenase		-1.65		1.07
SACE_1439	rpsT - 30S ribosomal protein S20		1.13		-1.12
SACE_1956	cvnD6 - ATP_GTP-binding protein		1.08		-1.01
SACE_6036	pyrH - uridylate kinase		1.06		-1.19
