# Supplementary material for: Integrated omics approaches provide strategies for rapid erythromycin yield increase in Saccharopolyspora erythraea
Source: Microb Cell Fact. 2016 Jun 3;15:93. doi: 10.1186/s12934-016-0496-5 (PMC4891893; doi:10.1186/s12934-016-0496-5)
Supplement: Supplementary file 12 — 10.1186/s12934-016-0496-5 Figures representing western blot analyses of constitutively over-expressed ilvB1 gene and mms operon (mmsOp). [file 12934_2016_496_MOESM12_ESM.pdf]

Additional file 12. Western blot analysis

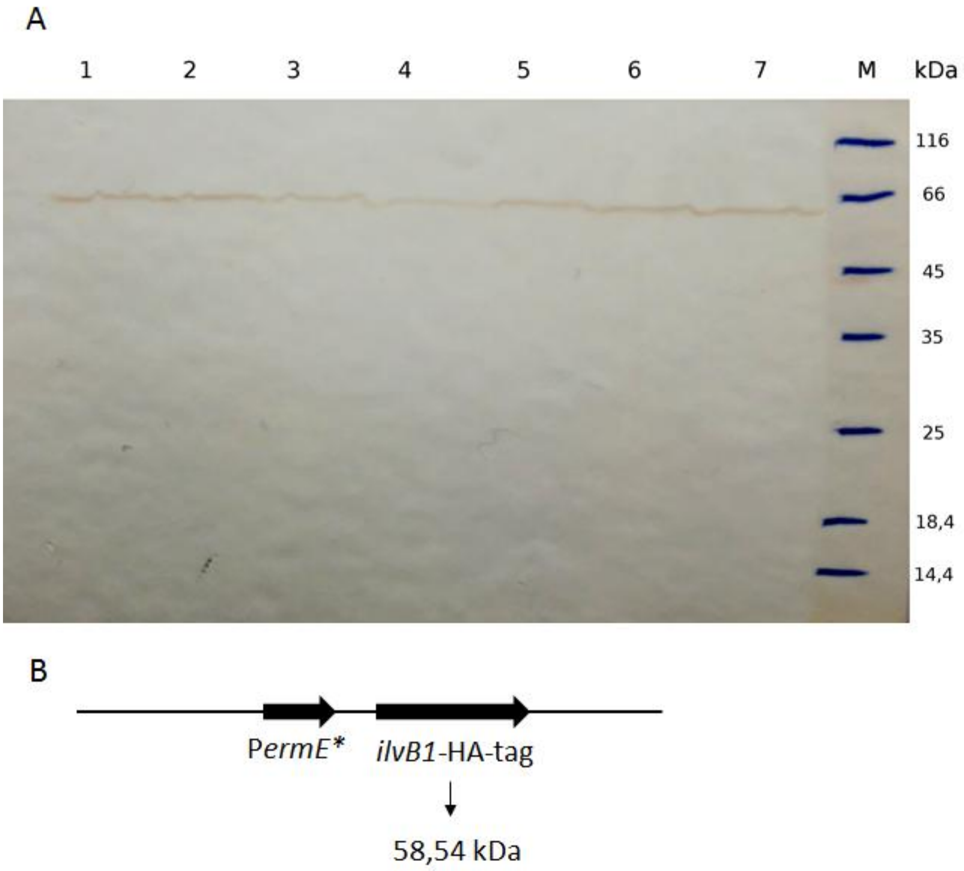

Figure 1: Western blot analysis and genomic context of *ilvB1* gene. A) Western blot analysis of *in trans* expression of 556 AA (lanes 1-7) *ilvB1*-HA-tag, M: molecular mass marker (their position are represented on the right side of the blot). Bands od apparent molecular mass of 58,54 kDa are observed in 7 independent 556 AA transformants of *S. erythraea* WT strain with expressed extra copy of *ilvB1* gene. B) Gene *ilvB1* (SACE_4565) is HA-tagged.

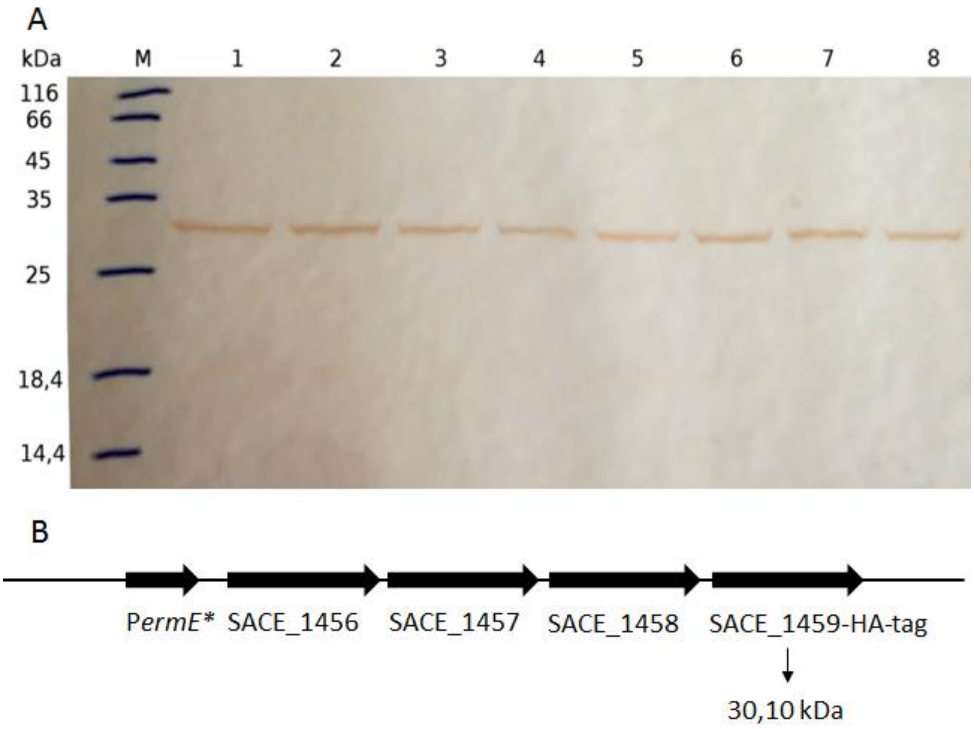

Figure 2: Western blot analysis and genomic context of *mms* operon. A) Western blot analysis of *in trans* expression of 295 AA (lanes 1-8) SACE_1459-HA-tag, M: molecular mass marker (their position are represented on the left side of the blot). Bands od apparent molecular mass of 30,10 kDa are observed in 8 independent 295 AA transformants of *S. erythraea* WT strain with expressed extra copy of SACE_1459 gene. B) Genetic map of *mmsOp1* (SACE_1456-59), the last gene in operon (SACE_1459) was HA-tagged.

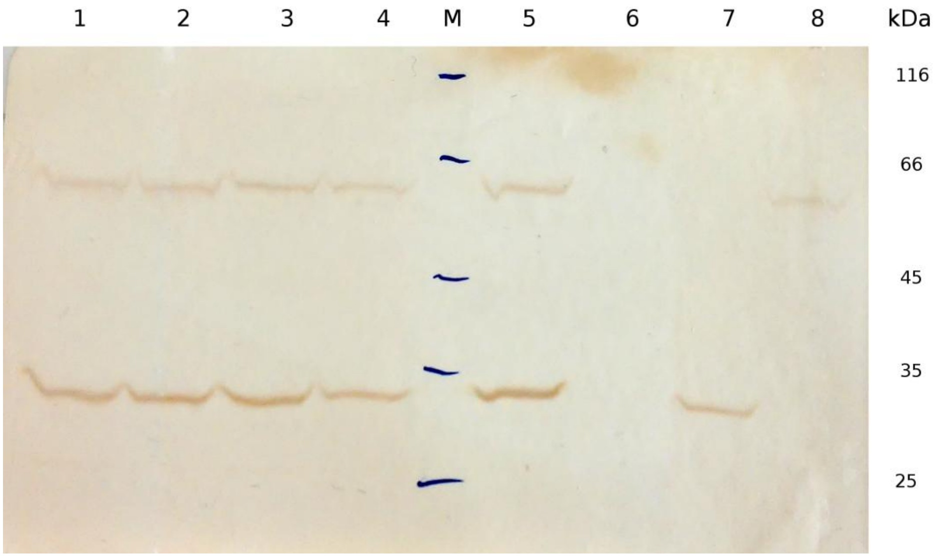

Figure 3: Western blot analysis of *in trans* expression of 295 AA, SACE_1459-HA-tag and 556 AA, *ilvB1*-HA-tag. 1-3: WT+pABE89, 4: WT+pABE88, M: molecular mass marker (their position are represented on the right side of the blot); 5: WT+pABE88; 6: negative control, strain WT; 7: WT+pABE87, 8: WT+pABE62 (bands of apparent molecular mass of 30,10 kDa and 58,54 kDa are observed).
