# Supplementary material for: Integrated omics approaches provide strategies for rapid erythromycin yield increase in Saccharopolyspora erythraea
Source: Microb Cell Fact. 2016 Jun 3;15:93. doi: 10.1186/s12934-016-0496-5 (PMC4891893; doi:10.1186/s12934-016-0496-5)
Supplement: Supplementary file 13 — 10.1186/s12934-016-0496-5 A detailed description of selected methods: preliminary qPCR analysis of key erythromycin related genes, ABE1441 strain sequencing and read mapping metrics, proteomic analysis (2-D electrophoresis, spectral counting), oligonucleotide primers used in genetic engineering of S. erythraea. [file 12934_2016_496_MOESM13_ESM.pdf]

Additional file 13 – Detailed description of selected methods

Preliminary qPCR analysis of key erythromycin related genes

Preliminary qPCR analysis was performed on dense time point series (15-20 time points) of three independent fermentations of WT in HP strains using selected marker genes, related to erythromycin biosynthesis.

Table: Primers and probes used for preliminary and microarray validation analysis. Gene ID, short name, description, primer and probe sequences (5’-3’) and assay efficiencies are shown. All probes were labelled with FAM and MGB.

Gene ID	ShortName	Description	Primer/probe sequence	Efficiency
SACE_0713	eryK	cytochrome P450 Erythromycin B/D C-12 hydroxylase	F: TCCTGGCCGAGGTCGA<br>R: GCAGCGCGGTGGAGAA<br>P: TTGGCCGCCTCCTCG	93%
SACE_0721	eryAI	EryAI Erythromycin polyketide synthase modules 1 and 2	F: TCGTGCACGGCCTGAT<br>R: CGCGTGCACCACACC<br>P: ACGTCGCCCTGCTCG	93%
SACE_1801	sigA	RNA polymerase major sigma factor	F: TCTCGCTGGACCAGACCAT<br>R: CCGCCTCGGAGTCCTC<br>P: CAGCTCGGTGACTTCA	93%
SACE_0302	katA	catalase	F: CTCGCGGGCACCGA<br>R: CCGCCTTGATGGTGTTCCA<br>P: CCGACCACCACATCCG	93%
SACE_2892	ebrB	multidrug resistance efflux protein	F: CGCCGTCCTGCTGCT<br>R: CCGACGACCACCACGAG<br>P: CCGACACCGCCTCCAG	85%
SACE_5639	mutB	methylmalonyl-CoA mutase (MCM)	F: CCAACGCCCTGGACGAG<br>R: GCACCAGCTGGGTGTTG<br>P: CTGCCGACCGACTTC	97%
SACE_5599	lmbU	conserved hypothetical protein present in several antibiotic biosynthetic clusters	[1]	
16S rRNA				

S. erythraea ABE1441 sequencing and read mapping metrics

a) Read mapping summary*

Total reference length	8.212.805
GC contents in %	71,15
Total read count	755.650
Not mapped read count	1.772
Not mapped reads - % of all reads	0,23
Mapped read count	753.878
Mapped reads - % of all reads	99,77
Mean mapped read length	389,45
Total mapped read length	293.601.369
Total mapped consensus length	8.197.582
Non-specific matches - read count	7.725
Non-specific matches - % of all mapped reads	1,02

b) Coverage statistics*

Minimum coverage	0
Maximum coverage	154
Average coverage	35,66
Standard deviation	11,63
Minimum excl. zero coverage regions	1
Average excl. zero coverage regions	35,73
Standard deviation excl. zero coverage regions	11,54

* The data was abstracted from CLC Genomics Workbench's Mapping Report.

Proteomic analysis

At selected time points 4 ml of bioprocess broth were centrifuged and the cell pellet was washed twice with 50 mM Tris-HCl, pH = 7.2. To prepare cell extract the cell pellet was resuspened in 400 uL of extraction buffer containing 7 M urea, 2 M thiourea, 4 % (w/v) CHAPS, 40 mM Tris, 65 mM DTT, protease inhibitor cocktail (Roche) - two tablets/10 mL of buffer and disrupted by sonification - four times, 15 s each with 30 s intervals for cooling the mixture on ice. The cell homogenate was centrifuged at 20000 g for 20 min at 4 °C. Protein concentration in the cell extract was determined using the method of Bradford [2].

Proteins were identified by LC-MS/MS and relative quantification of proteins in the cell extract was performed by two methods, 2-D electrophoresis [3] and spectral counting.

2-D electrophoresis

For 2-D electrophoresis the cell extracts were purifed using using 2-D Clean Up kit (GE Healthcare) according to manufacturer's instructions. Then the samples (150 µg protein) were mixed with rehydration solution (7 M urea, 2 M thiourea, 2% [w/v] CHAPS, 2% [v/v] immobilised pH gradient [IPG] buffer [pH 4-7], 18 mM dithiothreitol and a trace of bromophenol blue), and applied to 13-cm pH 4 to 7 IPG strips (GE Healthcare). After rehydration, the isoelectric focusing was carried out at 20 °C on a Multiphore II system (GE Healthcare). The following voltage programme was applied: 0–300 V (gradient over 1 min), 300 V (fixed for 1 h), 300–3500 V (gradient over 1.5 h) and 3500 V (fixed for 5 h). Prior to the second dimension of the 2-D electrophoresis, the IPG strips were equilibrated in sodium dodecyl sulphate (SDS) equilibration buffer (75 mM Tris HCl, pH 8.8, 6 M urea, 30% [v/v] glycerol, 2% [w/v] SDS and a trace of bromophenol blue), containing 1% dithiothreitol, for 15 min, and containing 4.8% iodoacetamide for an additional 15 min. The second dimension (SDS polyacrilamide gel electrophoresis) was carried out with the 12% running gels on a vertical SE 600 discontinuous electrophoretic system (Hoefer Scientific Instruments), at a constant 20 mA/gel for 15 min, and then at a constant 40 mA/gel until the bromophenol blue reached the bottom of the gels. The 2-D gels were stained with SYPRO Ruby (Invitrogen) and documented using a CAM-GX-CHEMI HR system (Syngene). Analysis of gel images (comparison of protein profiles between a wild type (WT) and industrial high producing (HP) strain of Saccharopolyspora erythraea at selected time points) was carried out using the 2-D Dymension software, version 2.02 (Syngene). Triplicate gels for each sample were matched to provide an average gel sample. The spots were revealed and quantified on the basis of their normalized volumes, defined as the spot volume divided by the total volume over the whole set of gel spots. Expression changes (fold changes) between WT and HP strain for particular time point were considered as significant when the intensity of the corresponding spots reproducibly differed by more than 1.5-fold in a normalized volume was statistically significant ($P<0.05$).

The differentially expressed proteins were cut from the gel and identified by mass spectrometry. LC-MS/MS analysis was performed as described below, with the only differences being shorter HPLC gradient (30 minutes) and database search. Database search was performed by a Sequest algorithm imbedded into Proteome Discoverer software (Version 1.3.0.339) (Thermo Scientific). Protein database, obtained from the *Saccharopolyspora erythraea* Genome Project Web site (http://jblseqdat.bioc.cam.ac.uk/gnmweb/files.html) was used for the search. Trypsin cleavage with maximum 2 missed cleavages was set as cleavage specificity, with methionine oxidation set as variable and cysteine carbamidomethylation as stable modification. Mass tolerances were set at 10 ppm for precursor and 0.8 Da for fragment ions. Decoy database search was performed with false discovery rate (FDR) set at 1% for peptide identifications.

Spectral counting

For quantification by spectral counting cell extract was separated by SDS-PAGE using a 12% Tris-Glycine precast gel (Lonza). Gel was stained by Commasie Brilliant Blue and whole protein lanes were cut into eight bands. Each gel band was destained in a solution of 25 mM ammonium bicarbonate and 50 % acetonitrile and prepared for mass spectrometry analysis. Destained gel bands were first reduced in the presence of 10 mM DTT in 25 mM ammonium bicarbonate (45 minutes at 56 °C). Reduction was followed by acetylation of cysteines by 55 mM iodoacetamide in 25 mM ammonium bicarbonate (30 minutes at room temperature). Gel pieces were washed with 25 mM ammonium bicarbonate dried and digested by trypsine (Promega) overnight at 37 °C. After digestion, peptides were extracted from the gel pieces by extraction solution (50 % acetonitrile / 5 % formic acid) and concentrated to 15 µl. LC-MS/MS analysis was performed with an EASY-nanoLCII HPLC unit (Thermo Scientific) coupled to an Orbitrap LTQ Velos mass spectrometer (Thermo Scientific). The peptide sample was first loaded on a C18 trapping column (Proxeon EASY-ColumnTM, 2 cm (length), 100 µm internal diameter, 5 µm 120 Å, C18-A1 beads) and then separated on a 10 cm long C18 PicoFritTM AQUASIL analytical column, (75 µm internal diameter, 5 µm 100 Å, C18 beads) (New Objective) using forward flushing. Peptides were eluted with a 90 minute linear gradient of 5-50 % solvent B (0.1 % formic acid in acetonitrile) at a flow rate of 300 nl/min. MS/MS spectra were obtained by fragmentation of the nine most intense precursor ions from the full MS scan. Dynamic exclusion was enabled with repeat count of 2 and 120 seconds exclusion time. The database search and quantification by spectral counting were performed using the MaxQuant proteomics software [4, 5] and S. erythraea protein database obtained from the Saccharopolyspora erythraea Genome Project Web site (http://jblseqdat.bioc.cam.ac.uk/gnmweb/files.html). Carbamidomethylation of cysteines was set as fixed modification, while methionine oxidation and N-terminal acetylation were set as variable modifications. Precursor and fragment mass tolerances were set at 6 and 20 ppm. Reversed database search was performed and false discovery rate (FDR), was set at 1 % for peptide and protein identifications.

Oligonucleotide primers used for genetic engineering of *S. erythraea*

Table with names and sequences of oligonucleotide primers used in this study

Name	Sequence	Amplified gene(s)	Amplicon lenght (bp)
bkdOp F	5'-AAAATCATATGTGTCCAGGAAAACGGCGCTCA-3'	SACE_3952-54	3590
bkdOp R	5'-AAATCTAGAACTACAACTCGCCGAGCGCGGTGATCG-3'		
mmsOp1 F	5'-AAAACATATGACCAACGAGCTTGGGCACTTCATCGG-3'	SACE_1456-59	5035
mmsOp1 R	5'-AAATCTAGATTCAGTGACCGGAGTGCTCGCGGACC-3'		
*mmsOp*1-HA tag R	5'-AAATCTAGATTCAGGCGTAGTCCGGGACGTCGTACGGGT AGTGACCGGAGTGCTCGCGGACC-3'		5062
ilvB1 F	5'-TTTTAACATATGCCGGGCGCTGACAACGAC-3'	SACE_4565	1709
ilvB1 R	5'-TTTTCTAGATTCACTTCGGCGATGTCCTCTCGGTCG-3'		
ilvB1-HA tag R	5'-TTTTCTAGATTCAGGCGTAGTCCGGGACGTCGTACGGGTACT TCGGCGATGTCCTCTCGGTCG-3'		1736
mmsOp2 F	5'- AAAAAACATATGGTGGCTACTACACAGGCCGACGAG-3'	SACE_4672-73	2946
mmsOp2 R	5'- AAAAATCTAGAATCAGAGGGCGGACGTGGCTTCGA-3'		

References:

1. Kirm B, Magdevska V, Tome M, Horvat M, Karnicar K, Petek M, Vidmar R, Baebler S, Jamnik P, Fujs S, et al: SACE_5599, a putative regulatory protein, is involved in morphological differentiation and erythromycin production in *Saccharopolyspora erythraea*. Microb Cell Fact 2013, 12:126.
2. Bradford MM: A rapid and sensitive method for the quantitation of microgram quantities of protein utilizing the principle of protein-dye binding. Anal Biochem 1976, 72:248-254.
3. Gorg A: Two-dimensional electrophoresis. *Nature* 1991, 349:545-546.
4. Cox J, Mann M: MaxQuant enables high peptide identification rates, individualized p.p.b.-range mass accuracies and proteome-wide protein quantification. Nat Biotechnol 2008, 26:1367-1372.
5. Cox J, Michalski A, Mann M: Software lock mass by two-dimensional minimization of peptide mass errors. *J Am Soc Mass Spectrom* 2011, 22:1373-1380.
