# Supplementary material for: Integrated omics approaches provide strategies for rapid erythromycin yield increase in Saccharopolyspora erythraea
Source: Microb Cell Fact. 2016 Jun 3;15:93. doi: 10.1186/s12934-016-0496-5 (PMC4891893; doi:10.1186/s12934-016-0496-5)
Supplement: Supplementary file 14 — 10.1186/s12934-016-0496-5 A table with description of strains and plasmids generated and used in this study and graphical representation of over-expressed operons/genes. [file 12934_2016_496_MOESM14_ESM.pdf]

Additional file 14

Table with description of strains and plasmids used in this study

Strain or plasmid	Descritpion	Source or reference
**Strains**		
WT	*S. erythraea* NRRL23338	
HP	*S. erythraea* ABE1441	Acies Bio
**Plasmids**		
pABE56	pSet152 derivative for the *PermE** controlled expression of *bkd* operon (SACE_3952-3954)	This study
pABE60	pSet152 derivative for the *PermE** controlled expression of *mmsOp1* (SACE_1456-1459)	This study
pABE61	pSet152 derivative for the *PermE** controlled expression of *ilvB1* (SACE_4565)	This study
pABE62	pSet152 derivative for the *PermE** controlled expression of *ilvB1*-HA tag	This study
pABE87	pSet152 derivative for the *PermE** controlled expression of *mmsOp1*-HA tag	This study
pABE88	pSet152 derivative for the *PermE** controlled expression of *ilvB1-mmsOp1*-HA tag	This study
pABE89	pSet152 derivative for the *PermE** controlled expression of *mmsOp1-ilvB1*-HA tag	This study
pABE95	pSet152 derivative for the *PermE** controlled expression of *mmsOp2* (SACE_4672-4673)	This study

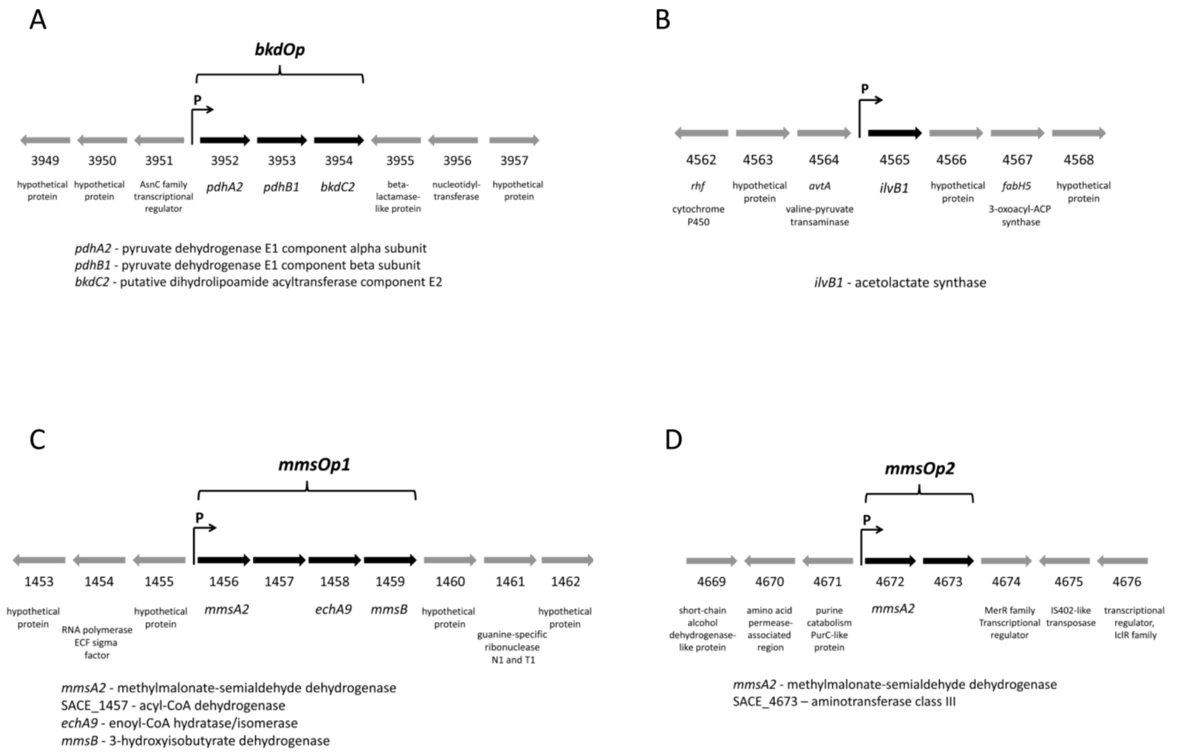

Figure: Graphical representation of the over-expressed operons/genes. Genes located close to operons/genes and their putative functions are indicated. SACE numbers are indicated under the arrows. All constructs were expressed under the control of strong constitutive promoter (P) *ermE**. **A)** *bkdOp* - operon encoding putative branched-chain ketoacid dehydrogenase subunits. **B)** *ilvB1* - putative acetolactate synthase. **C)** *mmsOp1* - operon encoding putative methylmalonate-semialdehyde dehydrogenase, acyl-CoA dehydrogenase, enoyl-CoA hydratase and 3-hydroxybutyrate dehydrogenase. **D)** *mmsOp2* - operon encoding putative methylmalonate-semialdehyde dehydrogenase homologue.
